# γ-Glutamylcysteine alleviates hypertension and vascular remodeling in SHR by suppressing oxidative stress through Keap1 S-glutathionylation

**DOI:** 10.1080/13510002.2026.2734676

**Published:** 2026-09-22

**Authors:** Jianzhen Lei, Yueru Kong, Chao Ye, Chuanyue Zhang, Yi Shen, Xin Wu, Qiuyue Wu, Ying Han, Min Chen, Yueqi Song, Qing Yan, Fen Zheng, Xinyi Xia

**Affiliations:** a Institute of Laboratory Medicine, Jinling Hospital, Affiliated Hospital of Medical School, State Key Laboratory of Analytical Chemistry for Life Science, Nanjing University, Nanjing, China; b College of Life Sciences, Nanjing Normal University, Nanjing, China; c Department of Basic Medicine, Wuxi School of Medicine, Jiangnan University, Wuxi, China; d Institute of Clinical Laboratory Science, Jinling Clinical Medical College, Nanjing University of Chinese Medicine, Nanjing, China; e Institute of Laboratory Medicine, Jinling Hospital, Nanjing Medical University, Nanjing; f Department of Hematology, Jinling Hospital, Affiliated Hospital of Medical School, Nanjing University, Nanjing, China; g The Affiliated Taizhou People's Hospital of Nanjing Medical University, Taizhou School of Clinical Medicine, Nanjing Medical University, Taizhou, China

**Keywords:** γ-Glutamylcysteine, hypertension, vascular remodeling, vascular smooth muscle cells, phenotypic switching, oxidative stress, Nrf2/Keap1, S-glutathionylation

## Abstract

**Objectives:**

Pathological arterial remodeling heavily depends on the aberrant proliferation and migration of vascular smooth muscle cells (VSMCs). This study explores how *γ*-Glutamylcysteine (*γ*-GC) regulates VSMC signaling to mitigate hypertensive structural damage.

**Methods:**

Spontaneously hypertensive rats (SHR) and Wistar-Kyoto (WKY) rats received intraperitoneal injections of *γ*-GC (100 mg/kg) every two days for 4 weeks. Blood pressure and arterial remodeling were evaluated. Primary aortic VSMCs were cultured for cellular mechanism analysis. To verify Nrf2 pathway requirement, the Nrf2 inhibitor ML385 was applied *in vitro* and via osmotic pumps *in vivo*.

**Results:**

*γ*-GC lowered systemic blood pressure and prevented arterial medial thickening in SHR, while normotensive controls remained unaffected. In SHR-VSMCs, *γ*-GC inhibited cell migration, proliferation, and phenotypic switching, and alleviated cellular oxidative stress. RNA-seq revealed significant enrichment of the Nrf2 signaling pathway. Mechanistically, *γ*-GC induced Keap1 S-glutathionylation, which disrupted the Keap1 and Nrf2 interaction and promoted Nrf2 nuclear translocation. Notably, Nrf2 inhibition with ML385 abolished the protective effects of *γ*-GC *in vivo* and *in vitro*.

**Discussion:**

*γ*-GC attenuates hypertensive arterial remodeling by modulating intracellular signaling, specifically through Keap1 S-glutathionylation-dependent Nrf2 activation. The Keap1/Nrf2 signaling axis is required for *γ*-GC to restore VSMC function, suggesting a targeted molecular mechanism for cardiovascular protection.

## Introduction

Hypertension, a long-term health issue marked by high blood pressure in the arteries, impacts around 1 billion individuals globally and is a major contributor to deaths from cardiovascular disease [1]. Substantial evidence indicates that hypertension results in dysfunction of target organs, including the heart, brain, and kidneys [2]. Vascular remodeling serves as a fundamental pathological process that induces functional or structural alterations in blood vessels, leading to hypertensive complications [3,4]. It is widely accepted that inhibiting pathological vascular remodeling is a key strategy for treating hypertension and its complications. Therefore, it is essential to understand the mechanisms underlying vascular remodeling in hypertension and to develop novel therapeutic approaches.

Blood vessel walls consist of the intima, media, and adventitia. Vascular smooth muscle cells (VSMCs) are primarily located in the media of arteries and play a critical role in maintaining vascular tone and regulating blood pressure [5,6]. VSMCs exhibit a quiescent and contractile phenotype under normal physiological conditions, while activated VSMCs switch to migratory, proliferative, and synthetic phenotypes under pathological conditions, and this process is termed phenotypic switching [7,8]. Phenotypic switching of VSMCs is not only the core mechanism of vascular remodeling but also forms the pathological basis of hypertensive complications [9]. Consequently, inhibiting VSMC migration and proliferation or preventing phenotypic switching of VSMCs is a key strategy for limiting vascular remodeling in hypertension.

Oxidative stress, resulting from an imbalance between oxidative and antioxidant processes, contributes to vascular remodeling in hypertension [10,11]. Excessive production of reactive oxygen species (ROS) is a key driver of oxidative stress [12]. Glutathione (GSH), which is the most common antioxidant found in cells, acts to clear ROS and uphold the redox balance inside cells [13]. Depletion of GSH or impaired synthesis exacerbates oxidative stress and mitochondrial dysfunction, thereby promoting vascular remodeling in hypertension [14,15]. Thus, enhancing GSH biosynthesis represents a promising therapeutic strategy for hypertension. However, the large concentration gradient of GSH, with millimolar levels intracellularly (0.5–10 mM) versus micromolar levels extracellularly (2–20 μM in plasma), poses a major barrier to direct exogenous supplementation [16].

*γ*-Glutamylcysteine (*γ*-GC), an immediate precursor of GSH, not only enters cells via transmembrane transport to facilitate GSH synthesis but also directly scavenges ROS [17,18]. Previous studies have reported that *γ*-GC exhibits a wide range of pharmacological effects, including neuroprotection [19], anti-inflammatory effects [20], treatment of age-related diseases [21], and the prevention of insulin resistance [22]. Crucially, recent studies from our laboratory have shown that *γ*-GC prevents the progression of atherosclerosis by inhibiting VSMC-derived foam cell formation [23].

Building on these findings, we hypothesized that *γ*-GC could serve as a novel therapeutic agent to alleviate hypertensive vascular remodeling. Specifically, we investigated whether *γ*-GC acts through a specific regulatory mechanism involving Keap1 S-glutathionylation to activate the Nrf2-mediated antioxidant defense system. To test this hypothesis, we utilized the spontaneous hypertensive rat (SHR) model [24] and primary VSMCs to evaluate the impact of *γ*-GC on systemic blood pressure and arterial structural integrity, while elucidating the underlying molecular signaling pathways.

## Materials and methods

### Reagents and antibodies

*γ*-Glutamylcysteine (*γ*-GC, CAS: 636-58-8, Cat. #HY-113402), ML385 (Cat. #HY-100523), buthionine sulfoximine (BSO, Cat. #HY-106376A), *N*-Ethylmaleimide (NEM, Cat. #HY-D0843) were obtained from Med Chem Express (Shanghai, China). BeyoClick™ EdU Cell Proliferation Kit (Cat. #C0078S), DCFH-DA probe (Cat. #S0033S), DHE probe (Cat. #S0064S), GSH and GSSG Assay Kit (Cat. #S0053), RIPA lysis buffer (Cat. #P0013E), and BCA assay kit (Cat. #P0009) were purchased from Beyotime (Shanghai, China). Assay kits for alanine aminotransferase (ALT, Cat. #C009-2-1), aspartate aminotransferase (AST, Cat. #C010-2-1), blood urea nitrogen (BUN, Cat. #C013-2-1), and serum creatinine (SCr, Cat. #C011-2-1) were obtained from Nanjing Jiancheng Bioengineering Institute (Nanjing, China). Details of the antibodies used in this study are provided in Table S1. Protein A/G PLUS-agarose (Cat. #SC-2003) was purchased from Santa Cruz Biotechnology (Santa Cruz, CA, U.S.A.).

### Animals and measurement of blood pressure

Eight-week-old adult male Wistar-Kyoto (WKY) rats and spontaneously hypertensive rats (SHR) were purchased from Vital River Laboratories Animal Technology Co., Ltd. (Beijing, China). According to the Guide for the Care and Use of Laboratory Animals (NIH publication, 8th edition, 2011), all the rats were placed in a stable environment at 25 °C and 40%–50% humidity. All animal experiments were approved by the Experimental Animal Use and Management Committee of Jinling Hospital affiliated with Nanjing University (approval No. DZYJSKT202508012002), and performed in the Animal Core Facility of Jinling Hospital and Nanjing Medical University.

Following a 7-day acclimatization and tail-cuff training period to minimize stress-induced blood pressure fluctuations, the 9-week-old SHR and WKY rats were randomly assigned to receive intraperitoneal injections of *γ*-GC (100 mg/kg) or vehicle (saline) every two days for four weeks. The *γ*-GC solutions were freshly prepared in saline before each administration to maintain stability. Blood pressure was recorded with a non-invasive tail-cuff system (NIBP, ADInstruments, Sydney, Australia) and the carotid catheter method. For the tail-cuff method, measurements were performed in conscious rats weekly for 4 weeks. The baseline characteristics of the rats used in our observations are shown in Table S3. For the carotid catheter method, rats were anaesthetized via intraperitoneal injection with a mixture of urethane (0.8 g/kg) and *α*-chloralose (40 mg/kg) after 4 weeks of *γ*-GC administration. A ventral neck incision was made to expose the common carotid artery, into which a PE-50 catheter filled with heparinized saline (10 U/mL heparin in 0.9% NaCl) was inserted for continuous blood pressure monitoring via a PowerLab data acquisition system (8SP, ADInstruments). Following blood pressure measurements, blood samples were collected and centrifuged to isolate serum. Serum levels of ALT, AST, BUN, and SCr were determined to evaluate the systemic safety of *γ*-GC. Finally, an overdose of pentobarbital (150 mg/kg) was administered intraperitoneally to euthanize the rats.

### Masson's staining

Masson's staining was performed to identify fibrosis within the arterial tissues. Aortic and mesenteric arteries were collected from SHR and WKY rats, fixed with 4% paraformaldehyde, embedded in paraffin, and sectioned at 5 μm. Sections were stained with Masson's trichrome kit and imaged using an Olympus light microscope (Olympus, Tokyo, Japan). Vascular remodeling was quantified by measuring the collagen fiber area, media thickness, lumen diameter, and the media thickness-to-lumen diameter ratio using ImageJ software (Bethesda, MD, U.S.A.).

### Cell culture and viability assays

Primary aortic vascular smooth muscle cells (VSMCs) were freshly isolated from the thoracic aortas of SHR and WKY rats. In short, the thoracic aorta was isolated, longitudinally opened, and the intimal layer was gently scraped with curved forceps to remove endothelial cells. The aorta was then cut into 2-mm pieces and placed evenly at the bottom of a cell culture flask. After a 6–8 hours adherence period, the tissue pieces were cultured in DMEM supplemented with 20% fetal bovine serum (FBS). VSMCs were passaged after 1 week and maintained in DMEM containing 10% FBS at 37 °C in a humidified atmosphere with 5% CO_2_.

Cell viability was assessed using a cell counting kit 8 assay (CCK8, Cat. #GK10001, GlpBio, Montclair, CA, USA). VSMCs were seeded in 96-well plates at a density of 10,000 cells per well. After adherence, cells were treated with increasing concentrations of *γ*-GC (0 to 160 μM) for 24 or 48 hours. Absorbance was measured at 450 nm.

### VSMC migration and proliferation assays

The VSMC migration was evaluated by boyden chamber assay and wound healing assay. For boyden chamber assay, Cells were placed in a serum-free medium within the upper section of a 24-well transwell insert featuring 8-μm pores (Cat. #CLS3422, Corning, NY, U.S.A.). The bottom chamber was filled with a complete medium that included 10% FBS. After incubating for 24 hours, cells that did not migrate on the upper surface were wiped away with a cotton swab. Migrated cells on the lower surface were fixed with 4% paraformaldehyde, stained with 0.5% crystal violet, and counted in five random fields using an optical microscope. For the wound healing assay, SHR and WKY VSMCs were seeded in 6-well plates and grown to 90% confluence. A sterile 200  μL pipette tip was used to scratch the wound. After incubating for 24 hours, the wound closure distance was examined using a light microscope.

VSMC proliferation was assessed by the EdU Cell Proliferation Kit. After placing SHR and WKY VSMCs in a 96-well plate, 10 μM EdU was introduced to the cells, which were then incubated for 2 hours. Cells were then fixed with 4% paraformaldehyde for 15 minutes at room temperature, washed with PBS, and stained with Click Additive Solution containing Hoechst 33342 for 30 minutes. EdU-positive cells were imaged and quantified using a fluorescence microscope (Nikon, Tokyo, Japan).

### DCFH-DA and DHE fluorescence analyzes

Intracellular ROS and superoxide levels were evaluated with the DCFH-DA probe (10 μM) and the DHE probe (10 μM), respectively. SHR-VSMCs were seeded in 6-well plates and then incubated with 10 μM DHE probe in dark for 1 hour followed by DHE fluorescence measurement (535 nm excitation and 610 nm emission), or stained with DCFH-DA probe in dark at 37 °C for 30 minutes for DCFH-DA fluorescence analyzes.

### Measurement of intracellular GSH levels

Intracellular total GSH and oxidized glutathione (GSSG) amounts were determined using the GSH and GSSG Assay Kit according to the manufacturer's protocol. Briefly, SHR-VSMCs were harvested and lysed in a specialized protein removal buffer. The supernatants were collected for the determination of total GSH and GSSG concentrations. The reduced GSH content was calculated by subtracting the GSSG concentration from the total GSH concentration.

### Immunoblotting

Total proteins were extracted from aorta tissue or primary aortic VSMCs from SHR and WKY rats using RIPA lysis buffer supplemented with a protease inhibitor cocktail. Protein concentrations were determined using a BCA assay kit. Equal amounts of protein (30 μg) were separated by 10% SDS-PAGE, transferred to a PVDF membrane (Cat. #IPVH00010, Millipore, Billerica, MA, USA), and blocked with 5% skim milk for 2 hours. It was then incubated with the appropriate primary antibody at 4 °C overnight. Following a TBST wash, the membrane was incubated with an HRP-linked secondary antibody for 1 hour. Visualization was done using an enhanced ECL immunoblotting system (Tanon, China). Band intensities were quantified using ImageJ software. *β*-actin served as the internal controls for total protein levels, while Lamin B was used as the internal control for nuclear protein levels.

### Quantitative reverse transcription PCR (qRT-PCR)

Total RNA was extracted from VSMCs and vascular tissues of SHR and WKY rats with Trizol reagent (Cat. #R401, Vazyme, China). A total of 1 μg of total RNA was reversed transcribed to cDNA by using Reverse Transcription Kit (Cat. #R233, Vazyme, China). Quantitative reactions were conducted using SYBR Green Mix (Cat. #Q712, Vazyme, Jiangsu, China) on the Step One Plus Real Time PCR system (Applied Biosystems, Foster City, CA, USA). Relative expression levels were calculated using the 2^−ΔΔCt^ method, with *β*-actin as the internal control. All primers used for qRT-PCR are provided in Supplementary Table S2.

### Immunofluorescence staining

SHR-VSMCs were incubated with 80 μM *γ*-GC for 24 hours, and fixed in 4% paraformaldehyde for 15 minutes. Cells were permeabilized with 0.1% Triton X-100 for 10 minutes, blocked with 5% BSA for 1 hour, and incubated overnight at 4 °C with anti-Nrf2 primary antibody. After washing with PBS, cells were incubated with secondary antibodies labeled with Cy3 for 1 hour at room temperature. Nuclei were counterstained with DAPI. Immunostaining was visualized using fluorescence microscope (Nikon, Japan).

### Nrf2 inhibition

To verify the essential role of Nrf2 both in vitro and in vivo, the specific Nrf2 inhibitor ML385 was employed. For *in vitro* experiments, primary aortic VSMCs were pre-incubated with ML385 (2 μM) for 24 hours before the addition of *γ*-GC to evaluate the dependency of antioxidant effects on the Nrf2 pathway. For *in vivo* experiments, SHR were anaesthetized with sodium pentobarbital (50 mg/kg, i.p.) for the subcutaneous implantation of ALZET osmotic pumps (Cat. #2ML4, Durect Corporation, CA, USA) in the mid-scapular region. ML385 was dissolved in a vehicle of 50% DMSO and 50% PEG400 at a concentration of 25 mg/mL, providing a continuous dose of approximately 5 mg/kg/day for 4 weeks. This dosage was selected based on previous studies successfully employing continuous ML385 infusion in rodents [25,26], with appropriate dose translation for rats. Control groups received pumps filled with the equivalent vehicle. This sustained delivery method was utilized to ensure stable Nrf2 inhibition and minimize stress-induced blood pressure fluctuations throughout the experimental period.

### Co-immunoprecipitation and determination of S-Glutathionylation of Keap1

SHR-VSMCs were lysed in the ice-cold IP lysis buffer supplemented with protease inhibitor cocktail. Lysates were centrifuged (15,000 × g) at 4 °C for 15 minutes. Proteins were immunoprecipitated with indicated antibodies (0.5 μg) at 4 °C overnight. Protein A/G PLUS-agarose beads were then added and incubated with immunocomplexes for another 4 hours and washed four times with cold lysis buffer. The samples were then analyzed by immunoblotting.

For detection of S-glutathionylated Keap1, SHR-VSMCs were lysed in the IP lysis buffer supplemented with NEM (10 mM). Cell lysate supernatants were treated with anti-Keap1 antibody, purified using Protein A/G PLUS-agarose beads, and then resolved by SDS-PAGE under non-reducing conditions. The gels were immunoblotted with anti-Glutathione and anti-Keap1 antibodies.

### RNA-seq analysis

SHR-VSMCs were treated with vehicle (PBS) or *γ*-GC (80 μM) for 24 hours, followed by extraction of total RNA. The NanoDrop 2000 spectrophotometer and Agilent 2100 Bioanalyzer (Thermo Fisher Scientific, MA, USA) were used to evaluate RNA quality and quantity. Following the elimination of ribosomal RNA, the library was created using the Illumina Truseq RNA sample preparation Kit (Illumina Inc., CA, USA). Sequencing of the library took place on an Illumina HiSeq 4000 platform (Illumina Inc., San Diego, CA, USA).

### Molecular docking

The 3D structure of *γ*-GC (ID:123938) was downloaded from PubChem database (https://pubchem.ncbi.nlm.nih.gov/), and converted to PDBQT format using Open Babel 2.4.1 software. Crystal structures of Nrf2 (ETGE, ID:7ECA) and Keap1 (ID:5CGJ) were obtained from Protein Data Bank database (https://www.rcsb.org/), Proteins were prepared using PyMOL software (version.2.2.0) by removing water molecules and ligands, adding polar hydrogens, and performing energy minimization.

Protein-ligand docking of *γ*-GC to Nrf2 (ETGE) and Keap1 were performed with Autodock 4.2.4 software. Protein-Ligand Interaction Profiler (PLIP; https://plip-tool.biotec.tu-dresden.de/) was used to identify the non-covalent interactions between *γ*-GC and Keap1.

### Statistical analysis

All statistical analyzes were performed using GraphPad Prism 9 software (San Diego, CA, USA). Data are presented as the mean ± SD from at least three independent experiments. Prior to analysis, the normality of the data distribution and the homogeneity of variance were evaluated utilizing the Shapiro–Wilk test and the Brown–Forsythe test, respectively. For normally distributed data with equal variance, differences between two independent groups were analyzed using an unpaired two-tailed Student's *t*-test, whereas multiple comparisons were evaluated via one-way or two-way ANOVA followed by Tukey's post hoc test. If the data failed the normality test, the Kruskal‒Wallis test followed by Dunn's multiple comparisons test was employed. For data presenting unequal variances, the Brown–Forsythe ANOVA followed by Dunnett's post hoc test was applied. *P-*value < 0.05 was considered statistically significant.

## Results

### γ-GC does not affect the viability of VSMCs

VSMCs are the major cellular components in the vascular media and are closely related to blood pressure [5]. We evaluated the cytotoxicity of *γ*-GC on primary aortic VSMCs isolated from SHR (SHR-VSMCs) and WKY rats (WKY-VSMCs). To assess the effect of *γ*-GC on VSMC viability, cells were treated with different concentrations of *γ*-GC (20, 40, 80, and 160 μM) for 24 or 48 hours. The results of the CCK-8 assay showed that *γ*-GC treatment for 24 and 48 hours did not affect the cell viability of WKY-VSMCs (Figure S1A-B) and SHR-VSMCs (Figure S1C-D) at doses ranging from 0 to 80 μM. However, a higher concentration of 160 μM *γ*-GC significantly reduced the viability of WKY-VSMCs at both time points and of SHR-VSMCs after 48 hours. Consequently, *γ*-GC concentrations of 0, 40, and 80 μM were used in subsequent experiments.

### γ-GC reduces blood pressure and attenuates vascular remodeling in SHR but not WKY rats

**Figure 1.**
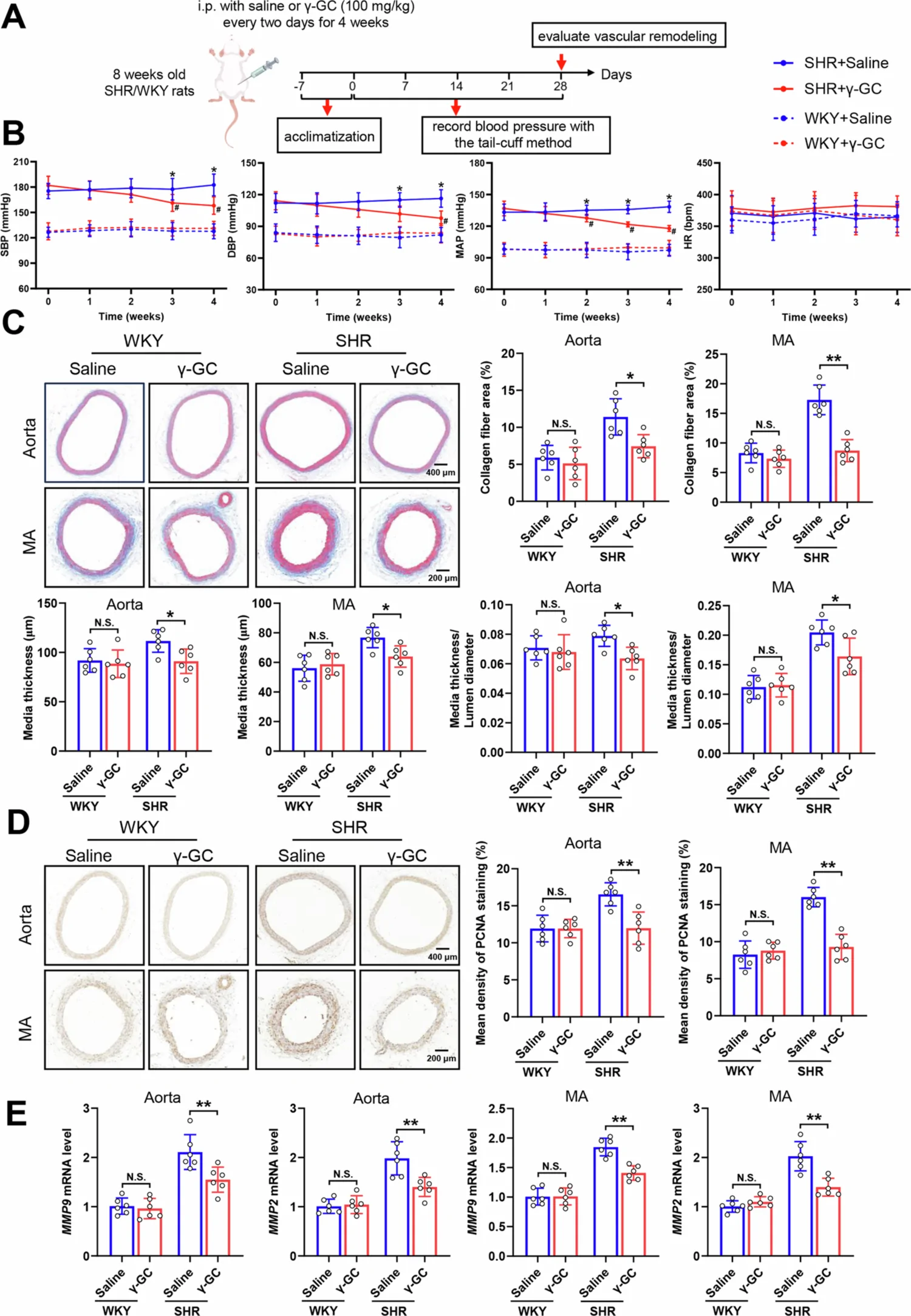
*γ*-GC reduces arterial blood pressure and attenuates vascular remodeling in SHR but not in WKY rats. (A) Schematic diagram showing the protocol of *γ*-GC intraperitoneal injection (100 mg/kg, every 2 days for 4 weeks) and blood pressure measurements in SHR and WKY rats. (B) Systolic blood pressure (SBP), diastolic blood pressure (DBP), mean arterial pressure (MAP) and heart rate (HR) were measured once a week in SHR and WKY rats with the tail-cuff method. Data are mean ± SD. *N* = 6 per group. *P < 0.05 for the SHR + γ-GC group (red solid line) vs the SHR + Saline group (blue solid line) at the corresponding week; ^#^ *P* < 0.05 for the SHR + γ-GC group (red solid line) vs its 0-week baseline. (C) Images of Masson's staining of the aorta and mesenteric arteries (MA) from SHR and WKY rats. Bar graph showing the Masson’s staining analysis for the collagen fiber area, medial thickness, and the ratio of medial thickness to lumen diameter in the aorta and MA. (D) Images of PCNA immunohistochemistry (brown color) of the aorta and MA from SHR and WKY rats. Bar graph showing the relative values of the PCNA immunohistochemistry analyzes. (E) The relative expression levels of *MMP9* and *MMP2* mRNA in SHR and WKY rat aorta and MA were measured by qPCR. Data are mean ± SD. *N* = 6 per group. *P < 0.05, **P < 0.01 vs indicated control. B were analyzed by two-way ANOVA followed by Tukey's post hoc test. C-E were analyzed by one-way ANOVA followed by Tukey's post hoc test.

To evaluate the effects of *γ*-GC on rat blood pressure, we performed intraperitoneal injection of *γ*-GC (100 mg/kg) or saline every two days for four weeks in SHR and WKY rats, the experimental flow chart is shown in Figure 1A. The blood pressure and heart rate of the rats were recorded weekly with the tail-cuff method. We found that *γ*-GC administration significantly reduced mean arterial pressure (MAP) in SHR after two weeks, and systolic or diastolic blood pressure after three weeks. Notably, *γ*-GC did not affect blood pressure in WKY rats (Figure 1B). Meanwhile, we detected SHR and WKY rats blood pressure with the carotid catheter method and obtained consistent results with those from the tail-cuff method (Figure S2). Consistently, *γ*-GC exerted a negative regulatory effect on blood pressure in hypertensive rats without affecting normal blood pressure.

Vascular remodeling is a central pathophysiological process in hypertension [27]. We examined whether administration of *γ*-GC affects rat vascular remodeling. Masson's staining results revealed that *γ*-GC obviously decreased the collagen fiber area, the media thickness, and the ratio of media thickness to lumen diameter in the aorta and mesenteric artery (MA) of SHR, but had no effect on those of WKY rats (Figure 1C and Figure S3A-B). Furthermore, to evaluate the systemic safety profile of *γ*-GC, we monitored rats body weight and measured serum biochemical markers for liver and kidney function. Comparatively, *γ*-GC treatment did not induce adverse changes in rats body weight or serum levels of ALT, AST, BUN, and SCr, nor did it have any discernable effect on rat kidney and liver histology (Figure S4). The abnormal proliferation and migration of VSMCs are the leading causes of vascular remodeling. Therefore, we next examined the expression of proliferating cell nuclear antigen (PCNA), a marker of cell proliferation, and matrix metalloproteinases (MMP2, MMP9), which play a pivotal role in cell migration [28]. The results of immunohistochemistry showed that PCNA expression was decreased after administration of *γ*-GC in the aorta and MA of SHR (Figure 1D). Meanwhile, qPCR results showed that the mRNA levels of *PCNA*, *MMP2,* and *MMP9* were decreased in the arteries of SHR following administration of *γ*-GC (Figure 1E and Figure S3C-D). Consistently, *γ*-GC had no effect on vascular remodeling in WKY rats (Figure 1C–E). Taken together, these results suggest that *γ*-GC reduces blood pressure in SHR by attenuating vascular remodeling.

### γ-GC inhibits migration, proliferation, and phenotypic switching in SHR-VSMCs

**Figure 2.**
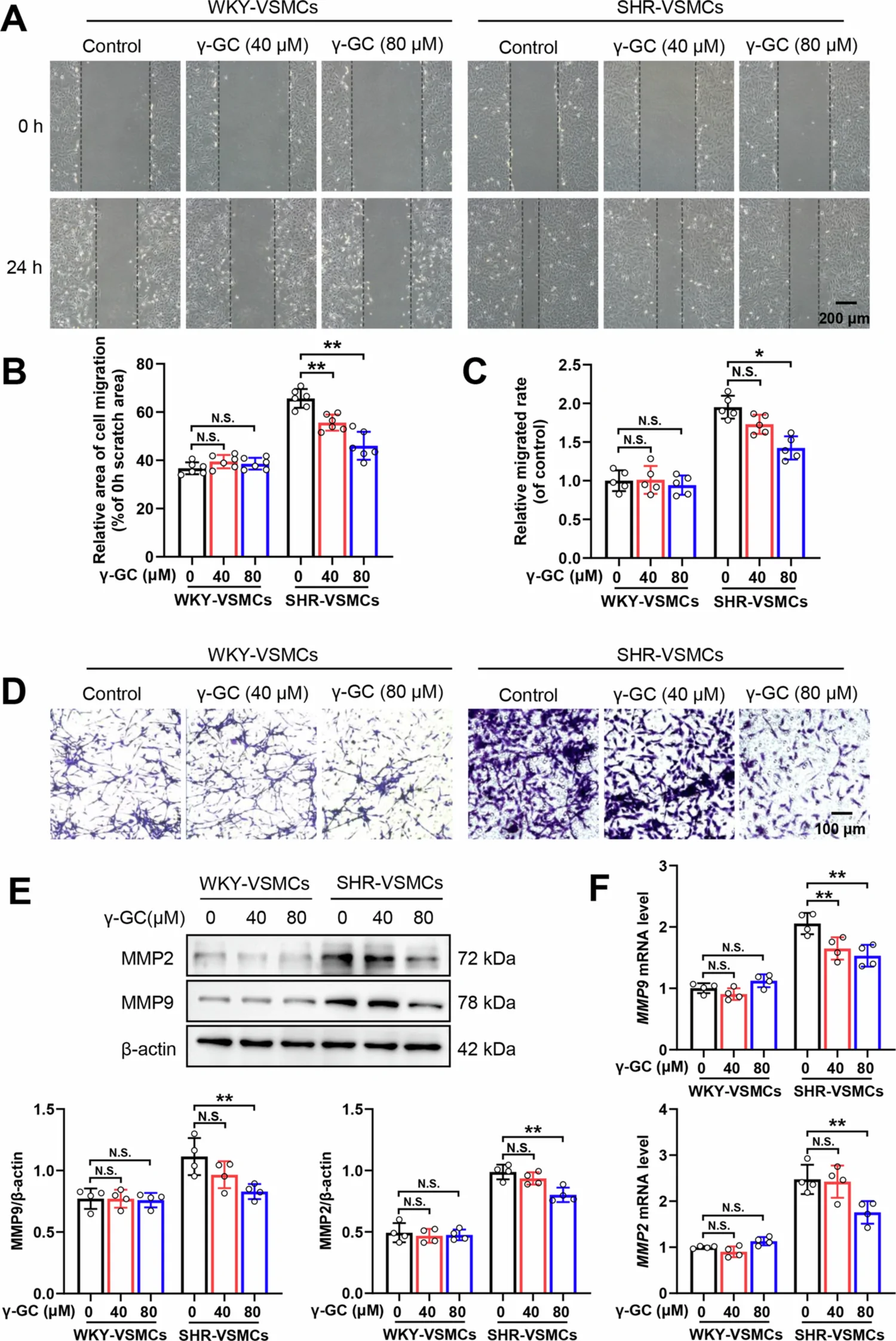
*γ*-GC inhibits cell migration in SHR-VSMCs but not in WKY-VSMCs. Primary SHR-VSMCs and WKY-VSMCs were treated with PBS or different concentrations of *γ*-GC (40 or 80  μM). Wound healing assay (A–B) and Boyden chamber assay (C–D) were performed to determine VSMC migration. (E) The expression levels of cell migration-related proteins MMP2 and MMP9 were examined by immunoblotting. (F) The relative expression levels of *MMP9* and *MMP2* mRNA were measured by qPCR. Data are mean ± SD. *N* = 6 per group in A and B, *N* = 5 per group in C and D, *N* = 4 per group in E and F. *P < 0.05, **P < 0.01 vs indicated control. A-F were analyzed by one-way ANOVA followed by Tukey's post hoc test.

**Figure 3.**
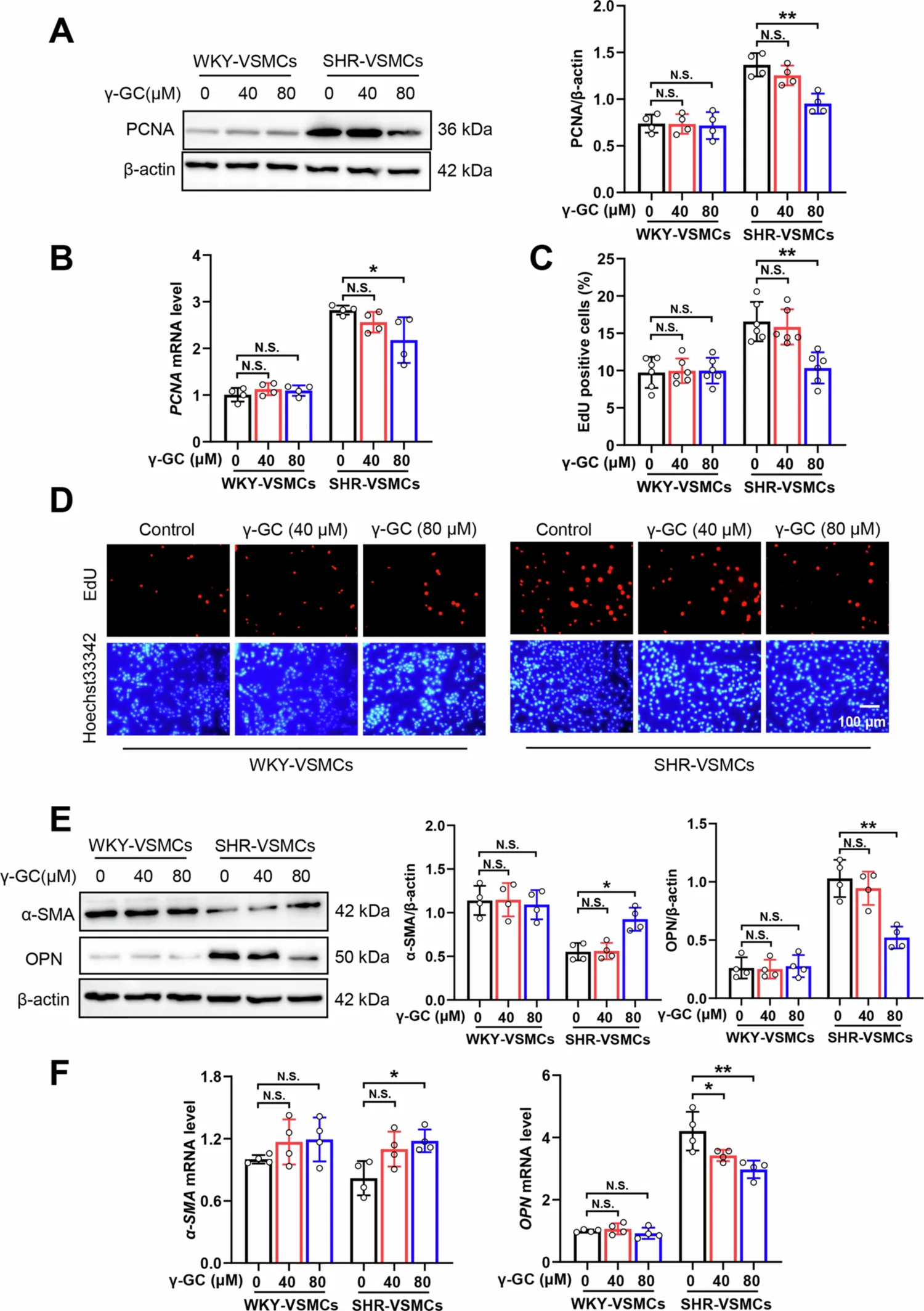
*γ*-GC inhibits cell proliferation and phenotypic switching in SHR-VSMCs but not in WKY-VSMCs. Primary SHR-VSMCs and WKY-VSMCs were treated with PBS or different concentrations of *γ*-GC (40 or 80 μM). (A) The expression levels of the cell proliferation-related protein PCNA were examined by immunoblotting. (B) The relative expression levels of *PCNA* mRNA were measured by qPCR. (C, D) Cell proliferation was examined by EdU fluorescence assay. (E) The expression levels of VSMC contractile phenotype protein *α*-SMA and VSMC synthetic phenotype protein osteopontin (OPN) were examined by immunoblotting. (F) The relative expression levels of *α-SMA* and *OPN* mRNA were measured by qPCR. Data are mean ± SD. *N* = 4 per group in A, B, E, and F, *N* = 6 per group in C and D. *P < 0.05, **P < 0.01 vs indicated control. A, C, E, and F were analyzed by one-way ANOVA followed by Tukey's post hoc test. B were analyzed by the Brown-Forsythe ANOVA followed by Dunnett's post hoc test.

The above *in vivo* experiments suggested that *γ*-GC exerts a protective effect against hypertension and vascular remodeling. Next, we investigated the effects of *γ*-GC on cell proliferation, migration, and phenotypic switching in VSMCs from SHR and WKY rats. To evaluate VSMC migration, we performed wound healing and Boyden chamber assays. We found that *γ*-GC treatment inhibited the migration of SHR-VSMCs (Figure 2A–D). Consistently, *γ*-GC decreased the expression of MMP2 and MMP9 in SHR-VSMCs (Figure 2E–F). Additionally, the results of the EdU assay indicated that *γ*-GC suppressed the proliferation of SHR-VSMCs (Figure 3A–B). Meanwhile, immunoblotting and qPCR results demonstrated that *γ*-GC significantly repressed PCNA expression (Figure 3C–D). However, *γ*-GC had no significant effects on WKY-VSMCs migration (Figure 2A–F) or proliferation (Figure 3A–D). Phenotypic switching of VSMCs is a core process associated with cell proliferation and migration. Here, we examined the expression of the contractile phenotype marker *α*-SMA and the synthetic phenotype marker osteopontin (OPN) in VSMCs from SHR and WKY rats. Immunoblotting and qPCR results showed that *γ*-GC increased *α*-SMA expression and suppressed OPN expression in SHR-VSMCs, but had no significant effects on WKY-VSMCs (Figure 3E–F). Taken together, these results indicate that *γ*-GC inhibits cell migration, proliferation, and phenotypic switching in SHR-VSMCs.

### γ-GC suppresses oxidative stress in SHR-VSMCs

**Figure 4.**
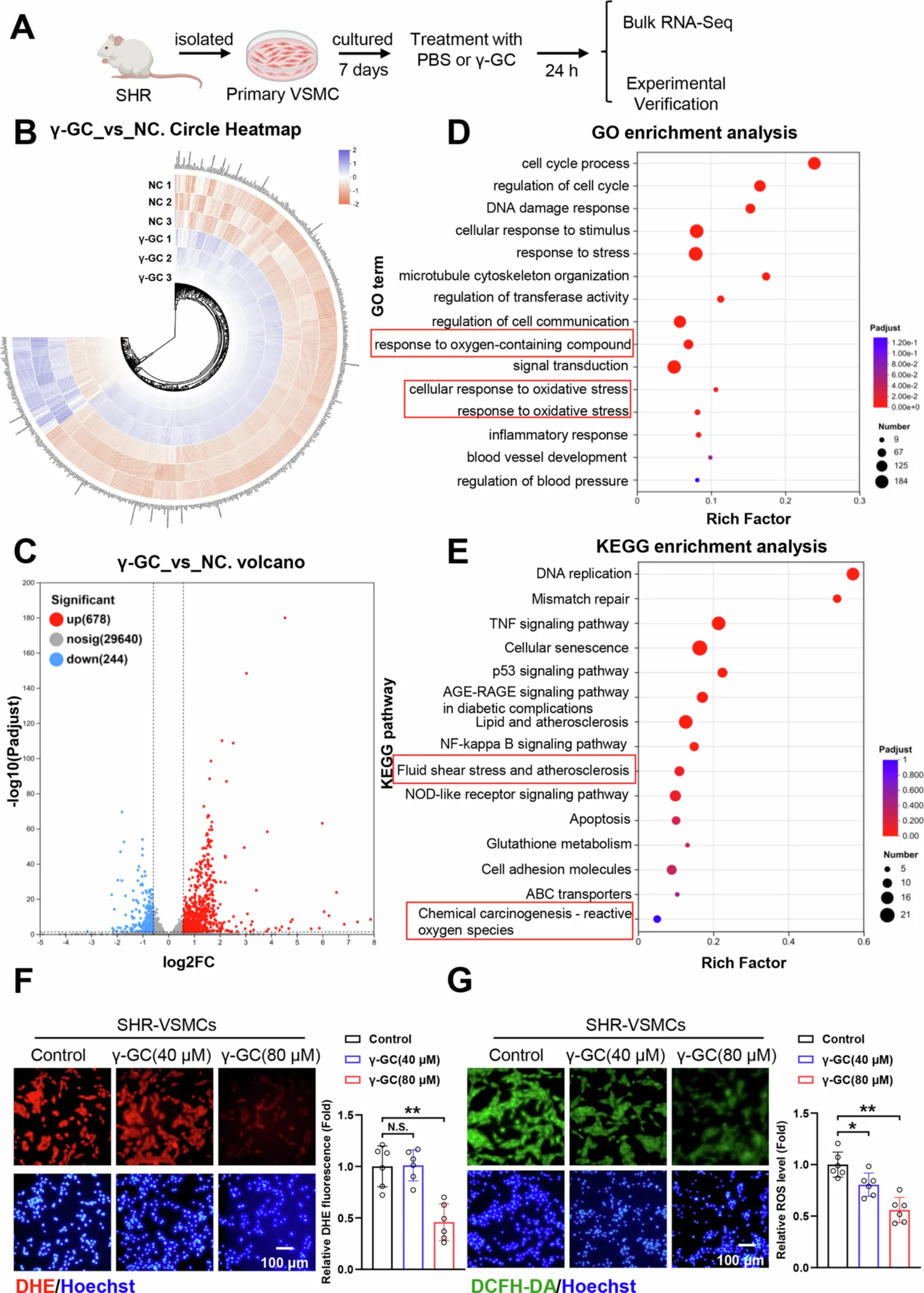
*γ*-GC suppresses oxidative stress in SHR-VSMCs. (A) Schematic diagram showing the experiment protocol. SHR-VSMCs were treated with PBS or *γ*-GC (80 μM) for 24 hours. Circle Heatmap (B) and Volcano plots (C) showed differential gene expression by RNA-seq. (D–E) Enrichment analysis of gene ontology (GO) analysis and Kyoto Encyclopedia of Genes and Genomes (KEGG) analysis. SHR-VSMCs were treated with PBS or different concentrations of *γ*-GC (40 or 80 μM). (F) The levels of superoxide anion were detected by DHE fluorescence staining. (G) The levels of reactive oxygen species (ROS) analyzed using the DCFH-DA probe. Data are mean ± SD. *N* = 6 per group in F and G. *P < 0.05, **P < 0.01 vs indicated control. F–G were analyzed by one-way ANOVA followed by Tukey's post hoc test.

To further investigate the underlying mechanism of *γ*-GC in VSMC phenotypic switching, we performed RNA-seq analysis in primary SHR-VSMCs with or without *γ*-GC (Figure 4A). A total of 17,192 genes and 31,892 expressed transcripts were detected (Figure 4B). Differential gene expression analysis was performed with DESeq2. As shown in Figure 4C, a total of 678 genes were up-regulated and 244 genes were down-regulated in *γ*-GC-treated SHR-VSMCs (*P*-value *<* 0.05, fold change *>* 1.5 or *<* 1/1.5). Gene ontology (GO) and Kyoto Encyclopedia of Genes and Genomes (KEGG) enrichment analyzes (Figure 4D–E) indicated that *γ*-GC treatment predominantly influenced pathways related to cell cycle arrest, which aligns well with its anti‑proliferative effect. Notably, we also observed a significant impact of *γ*-GC on oxidative stress‑associated pathways. Previous studies indicated that ROS overload and oxidative stress greatly contribute to VSMC phenotypic switching and vascular remodeling in hypertension [29]. Thus, we further examined whether *γ*-GC regulates VSMC phenotypic switching through redox signaling. DHE and DCFH-DA fluorescence staining confirmed that *γ*-GC significantly reduced the elevated levels of superoxide anions and total ROS in SHR-VSMCs (Figure 4F–G). In contrast, *γ*-GC did not significantly influence ROS levels in WKY-VSMCs, as evidenced by DHE and DCFH-DA staining (Figure S5). These results indicate that *γ*-GC reduces the elevated ROS levels in SHR-VSMCs but does not alter the basal ROS levels in normal WKY-VSMCs.

### γ-GC activates Nrf2 signaling to suppress oxidative stress in SHR-VSMCs

**Figure 5.**
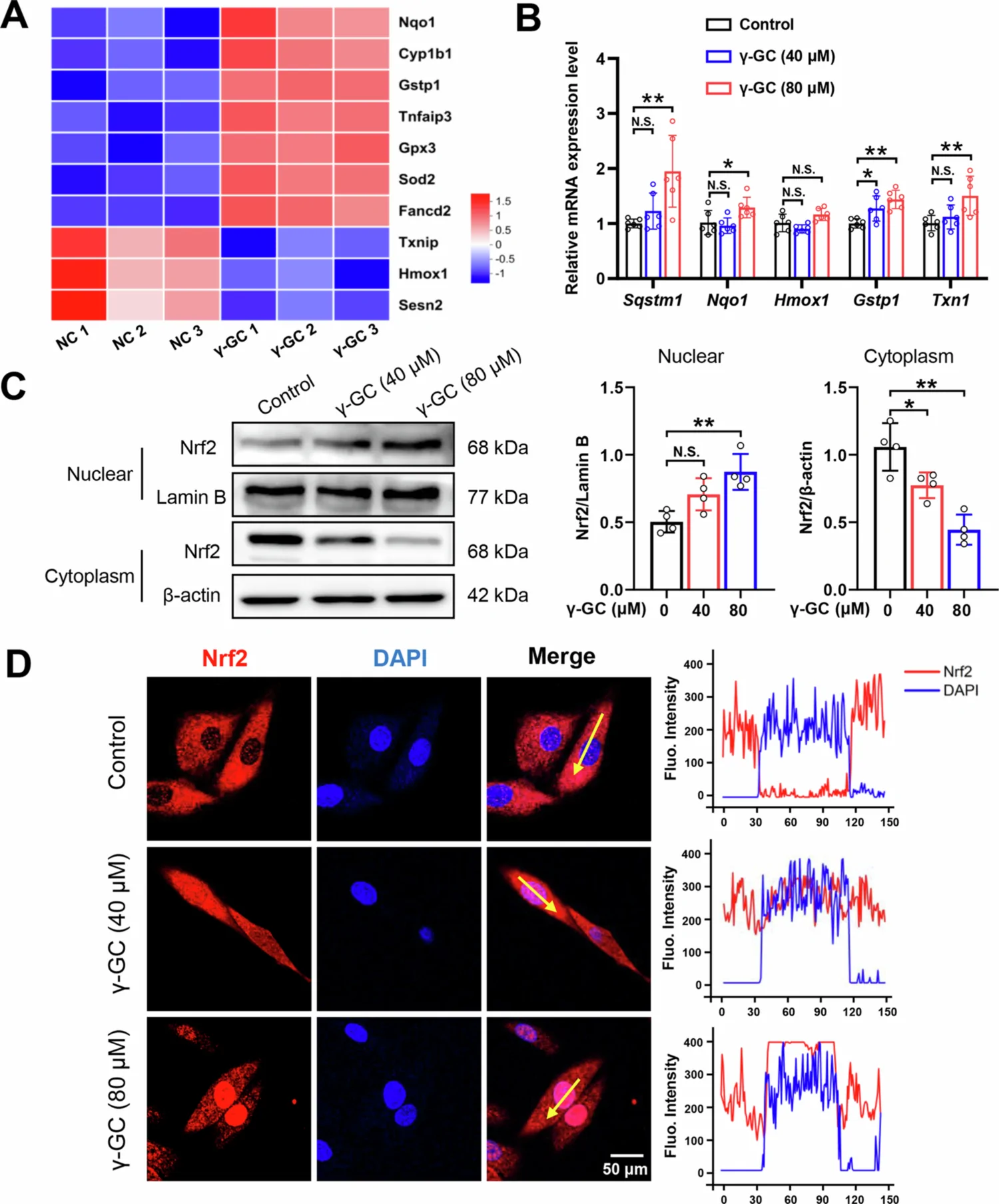
*γ*-GC promotes Nrf2 nuclear translocation in SHR-VSMCs. (A) Heatmap showed the expression of antioxidant-related genes by RNA-Seq. SHR-VSMCs were treated with PBS or different concentrations of *γ*-GC (40 or 80 μM). (B) qPCR detection of Nrf2-related genes mRNA levels. (C) Cell nuclear protein was extracted and Nrf2 expression in nucleus and cytoplasm were respectively assessed by immunoblotting. *β*-actin was used as an internal reference for cytoplasm, and Lamin B was used as an internal reference for the nucleus. (D) Nrf2 (red) distribution was detected by immunofluorescence assay (the nucleus was stained with DAPI). The yellow arrows indicate the representative lines used for fluorescence intensity profile analysis (right panels). Data are mean ± SD. *N* = 6 per group in B and D. *N* = 4 per group in C. *P < 0.05, **P < 0.01 vs indicated control. B–C were analyzed by one-way ANOVA followed by Tukey's post hoc test.

To further decipher the possible targets of *γ*-GC on VSMC oxidative stress, we next analyzed the RNA-seq data from SHR-VSMCs to identify antioxidant-related gene expression signatures. As shown in Figure 5A, the top ten genes related to oxidative stress were screened out, including *Nqo1*, *Cyp1b1*, *Gstp1*, *Tnfaip3*, *Gpx3*, *Sod2*, *Fancd2*, *Txnip*, *Hmox1,* and *Sesn2*. Notably, these genes are closely associated with the Nrf2 signaling pathway. Meanwhile, qPCR results confirmed that *γ*-GC upregulated the mRNA levels of Nrf2 downstream target genes, including *Sqstm1*, *Nqo1*, *Hmox1*, *Gstp1,* and *Txn1* in SHR-VSMCs (Figure 5B). Numerous studies have shown that Nrf2, as a transcription factor, plays a pivotal role in redox homeostasis [11,30]. We next explored whether *γ*-GC attenuates oxidative stress in VSMCs by activating Nrf2. SHR-VSMCs were treated with different concentrations of *γ*-GC, and nuclear and cytosolic Nrf2 protein levels were determined by immunoblotting. The results showed that nuclear Nrf2 levels markedly increased while cytosolic Nrf2 levels decreased following 80 μM *γ*-GC treatment in SHR-VSMCs (Figure 5C). Moreover, immunofluorescence analysis showed that *γ*-GC promoted Nrf2 nuclear accumulation in SHR-VSMCs (Figure 5D). Collectively, these results indicate that *γ*-GC activates Nrf2 and promotes its nuclear translocation in SHR-VSMCs.

### Inhibition of Nrf2 abolishes the protective effects of γ-GC against hypertension, vascular remodeling, and oxidative stress in SHR

**Figure 6.**
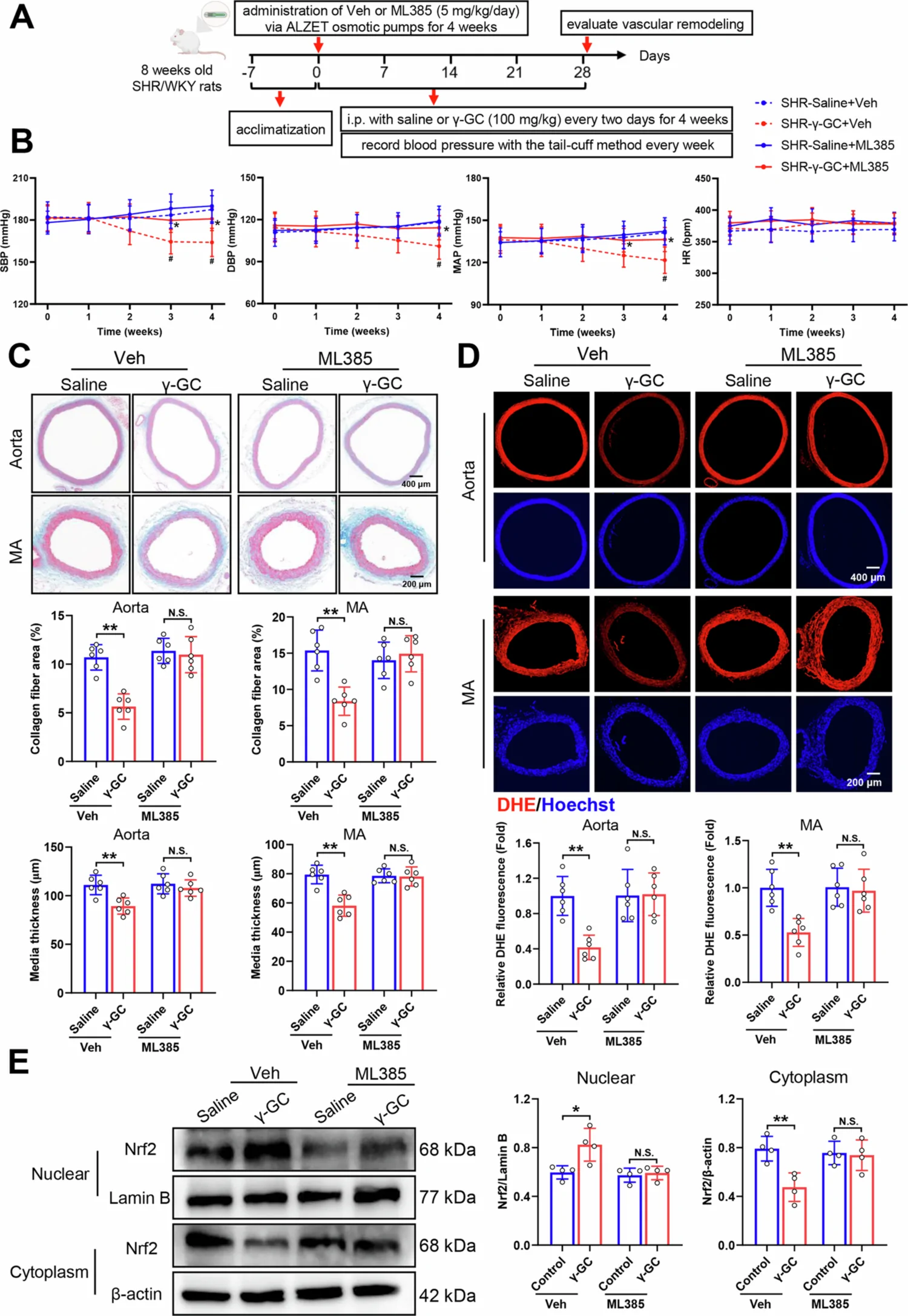
Nrf2 inhibition abolishes the effects of *γ*-GC on hypertension, vascular remodeling, and oxidative stress in SHR. (A) Experimental scheme for the administration of *γ*-GC (100  mg/kg, every 2 days for 4 weeks) and ML385 (5 mg/kg/day, via ALZET osmotic pumps) in SHR. (B) Systolic blood pressure (SBP), diastolic blood pressure (DBP), mean arterial pressure (MAP) and heart rate (HR) were measured once a week in SHR with the tail-cuff method. Data are mean ± SD. *N* = 6 per group. *P < 0.05 for the SHR-*γ*-GC + ML385 (red solid line) group vs the SHR-*γ*-GC + Veh (red dashed line) group at the corresponding week; ^#^ *P* < 0.05 for the SHR-*γ*-GC + Veh (red dashed line) group vs its 0-week baseline. (C) Images of Masson's staining of the aorta and mesenteric arteries (MA) from SHR. Bar graph showing the Masson's staining analysis for the collagen fibers area and the medial thickness in the aorta and MA. (D) Images of DHE fluorescence staining of the aorta and MA from SHR. (E) Aorta tissue nuclear protein was extracted and Nrf2 expression in nucleus and cytoplasm were respectively assessed by immunoblotting. *β*-actin was used as an internal reference for cytoplasm, and Lamin B was used as an internal reference for the nucleus. Data are mean ± SD. *N* = 6 per group in B–D, *N* = 4 per group in E. *P < 0.05, **P < 0.01 vs indicated control. B were analyzed by two-way ANOVA followed by Tukey's post hoc test. C–E were analyzed by one-way ANOVA followed by Tukey's post hoc test.

Subsequently, we used the Nrf2 inhibitor ML385 to determine whether the protective effects of *γ*-GC on hypertension and vascular remodeling are mediated by Nrf2. For the *in vivo* experiments, ALZET osmotic pumps were used to deliver ML385 (5 mg/kg/day) to SHR (Figure 6A), and its sustained *in vivo* efficacy was confirmed by the suppression of aortic Hmox1 and Nqo1 mRNA at week 4 (Figure S6A-B). The results demonstrated that ML385 significantly reversed the *γ*-GC-induced reduction in blood pressure (Figure 6B) and the inhibition of vascular remodeling (Figure 6C and Figure S6C-F) in SHR. Additionally, DHE fluorescence staining revealed that *γ*-GC suppressed ROS levels in the vascular wall, and this effect was significantly reversed by ML385 (Figure 6D). Immunoblotting results further confirmed that *γ*-GC promoted the nuclear translocation of Nrf2 in the vascular tissues of SHR, and this effect was blocked by ML385 (Figure 6E). Collectively, these *in vivo* findings indicate that Nrf2 activation is indispensable for the antihypertensive and antioxidant properties of *γ*-GC.

### Inhibition of Nrf2 reverses the effect of γ-GC on SHR-VSMC migration and proliferation

**Figure 7.**
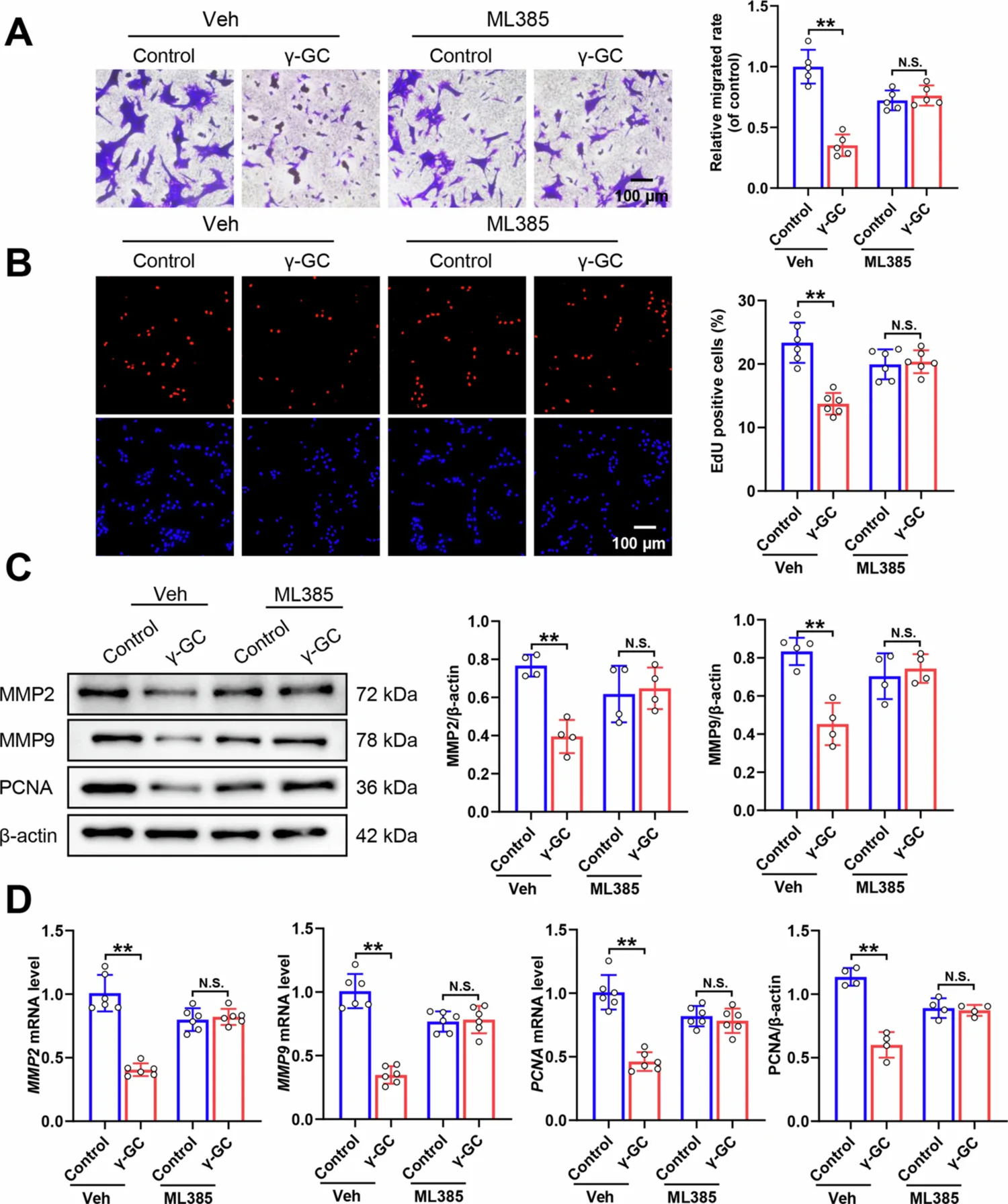
Nrf2 inhibition reverses the effects of *γ*-GC on SHR-VSMC proliferation and migration. SHR-VSMCs were pre-treated with ML385 (2 μM) followed by treatment with PBS or *γ*-GC (80  μM). Vehicle (Veh) served as a control. (A) Boyden chamber assay was performed to determine VSMC migration. (B) Cell proliferation was examined by EdU fluorescence assay. (C) The protein levels of MMP2, MMP9, and PCNA were examined by immunoblotting. (D) The relative mRNA levels of *MMP9*, *MMP2,* and *PCNA* mRNA were measured by qPCR. Data are mean ± SD. *N* = 5 per group in A. *N* = 6 per group in B and D. *N* = 4 per group in C. *P < 0.05, **P < 0.01 vs indicated control. A, B, and D were analyzed by one-way ANOVA followed by Tukey's post hoc test. In C, data for MMP2 were analyzed by the Brown-Forsythe ANOVA followed by Dunnett's post hoc test, while data for MMP9 and PCNA were analyzed by one-way ANOVA followed by Tukey's post hoc test.

To further confirm these findings in cells, we used ML385 to treat SHR-VSMCs. Boyden chamber (Figure 7A) and EdU assay (Figure 7B) results demonstrated that ML385 reversed the *γ*-GC-induced suppression of migration and proliferation in SHR-VSMCs. Meanwhile, the results of immunoblotting and qPCR showed that co-treatment with *γ*-GC and ML385 significantly increased the expression of MMP2, MMP9, PCNA, and OPN while decreasing *α*-SMA expression, compared with *γ*-GC treatment alone (Figure 7C–D and Figure S7). Collectively, these results indicate that *γ*-GC inhibits SHR-VSMC migration, proliferation, and phenotypic switching, thereby alleviating vascular remodeling by activating Nrf2.

### γ-GC activates Nrf2 by promoting Keap1 S-glutathionylation in SHR-VSMCs

**Figure 8.**
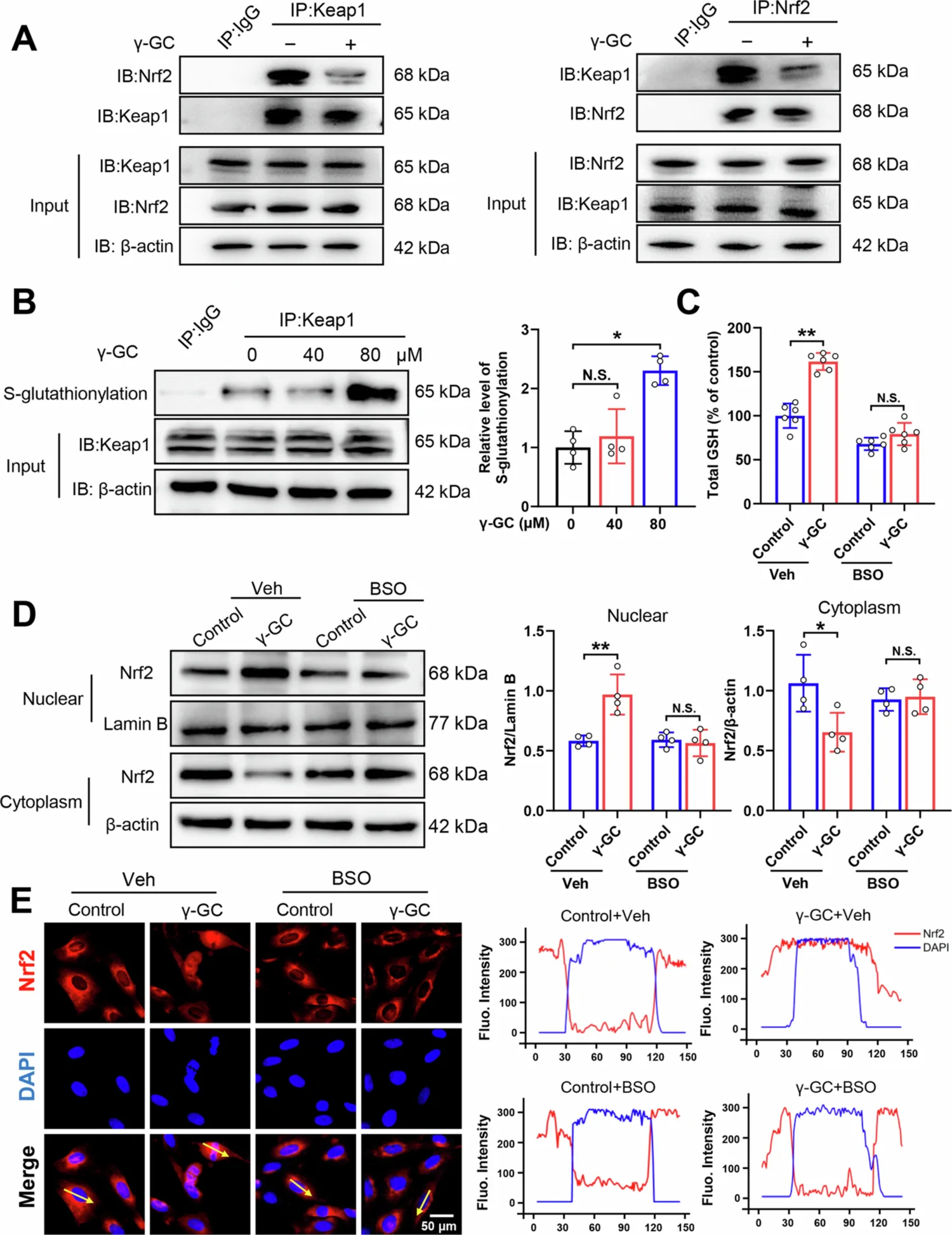
*γ*-GC activates Nrf2 by promoting Keap1 S-glutathionylation in SHR-VSMCs. (A) Co-immunoprecipitation assay was used to detect the interaction of endogenous Nrf2 with Keap1. (B) Keap1 S-glutathionylation levels measured by IP followed by non-reducing immunoblotting. SHR-VSMCs were pre-treated with BSO (1 mM) followed by treatment with PBS or *γ*-GC (80 μM). Vehicle (Veh) served as a control. (C) Total GSH levels were measured using the indicated commercialized kit. (D) Cell nuclear protein was extracted and Nrf2 expression in the nucleus and cytoplasm were respectively assessed by immunoblotting. *β*-actin was used as an internal reference for cytoplasm, and Lamin B was used as an internal reference for the nucleus. (E) Nrf2 (red) distribution was detected by immunofluorescence assay (the nucleus was stained with DAPI). Data are mean ± SD. *N* = 4 per group in C; *N* = 4 per group in others. *P < 0.05, **P < 0.01 vs indicated control. B were analyzed by the Kruskal–Wallis test followed by Dunn's multiple comparisons test. C and D were analyzed by one-way ANOVA followed by Tukey's post hoc test.

Keap1 functions as a repressor of the Nrf2 pathway by sequestering Nrf2 in the cytoplasm, and disruption of the Nrf2 and Keap1 complex is essential for Nrf2 activation [31]. Co-immunoprecipitation results demonstrated that *γ*-GC treatment reduced the interaction between Nrf2 and Keap1 in SHR-VSMCs (Figure 8A). Initial molecular docking suggested that *γ*-GC exhibited a binding energy of −3.24 kcal/mol with Nrf2 (ETGE) and −6.38 kcal/mol with Keap1 (Figure S8). However, the endogenous Nrf2-ETGE motif binds Keap1 with high affinity (5–20 nM, equivalent to approximately −11.3 kcal/mol) [32,33]. Thermodynamically, a ligand with this affinity (−6.38 kcal/mol) is roughly 4000-fold weaker than the native Nrf2 interaction and cannot competitively displace Nrf2 under physiological conditions. Given these thermodynamic constraints, we explored alternative mechanisms for the observed complex disruption.

As a redox-sensitive protein with reactive cysteine residues, Keap1 can undergo S-glutathionylation, a modification that promotes Nrf2 release and activation [34,35]. Non-reducing immunoblotting analysis using a monoclonal anti-glutathione antibody detected significantly increased S-glutathionylation of Keap1 in *γ*-GC-treated SHR-VSMCs (Figure 8B). Given that *γ*-GC is a GSH precursor, we hypothesized that it induces Keap1 S-glutathionylation via elevated intracellular GSH levels. To test this, we measured the intracellular GSH status and found that *γ*-GC treatment significantly increased total GSH levels and the GSH/GSSG ratio (Figure 8C and Figure S9). Furthermore, pretreatment with buthionine sulfoximine (BSO), a GSH synthesis inhibitor, abolished the enrichment of the intracellular GSH pool as well as the *γ*-GC-induced Nrf2 nuclear translocation, as evidenced by immunoblotting and immunofluorescence assays (Figure 8D–E). Taken together, these results demonstrate that *γ*-GC activates Nrf2 in SHR-VSMCs through promoting Keap1 S-glutathionylation.

## Discussion

Cardiovascular disease remains the leading cause of mortality and disability worldwide, with hypertension as its most significant risk factor [36,37]. Vascular remodeling, characterized by functional and structural alterations in blood vessels, plays a central role in the development and complications of hypertension. *γ*-GC acts as an immediate precursor of GSH and exerts potent antioxidant and anti-inflammatory effects [18]. Furthermore, *γ*-GC has been shown to be an effective and safe agent for augmenting GSH levels, overcoming the substantial barrier to the intracellular transport of exogenous GSH due to its steep concentration gradient [19]. However, its role in the cardiovascular system remains poorly understood. In our recent study, we demonstrated that *γ*-GC inhibits foam cell formation during early atherosclerotic lesion development [23]. Building on this, we investigated whether *γ*-GC also modulates vascular remodeling in hypertension. Here, we report for the first time that *γ*-GC reduces blood pressure in hypertensive rats (SHR) without affecting normotensive WKY rats. Furthermore, *γ*-GC attenuates hypertensive structural remodeling of the aorta and mesenteric artery, including increased collagen fiber area, media thickness, and the media-to-lumen ratio, without altering normal arterial architecture. This selective efficacy stems from their contrasting baseline redox states. In SHR, *γ*-GC activates Nrf2 to clear excessive ROS, reversing oxidative stress-driven remodeling and lowering blood pressure. Conversely, WKY rats maintain physiological redox homeostasis. Thus, augmenting their antioxidant capacity does not alter normal basal hemodynamics or vascular structure. Notably, *γ*-GC serves as a superior pharmacological strategy over direct GSH supplementation by bypassing membrane transport barriers and escaping feedback inhibition to efficiently restore the intracellular antioxidant reservoir. Despite its short plasma half-life, every-other-day dosing is justified because high tissue uptake and continuous intracellular recycling extend the functional half-life of *γ*-GC to approximately 48 hours [16,38]. Histological evaluation further confirmed the systemic safety of *γ*-GC, as no discernible structural abnormalities were observed in the liver or kidneys across all experimental groups.

VSMCs, the primary cellular component of the vascular media, play a key role in regulating arterial blood pressure [39]. Aberrant proliferation and migration of VSMCs represent central pathophysiological events in various cardiovascular diseases including hypertension [40]. In the current study, we isolated primary aortic VSMCs from SHR and WKY rats and found that low concentrations of *γ*-GC had no cytotoxic effect. Furthermore, wound healing, Boyden chamber, and EdU assay results demonstrated that *γ*-GC inhibited aberrant cell migration and proliferation in SHR-VSMCs but had no effect on WKY-VSMCs. Upregulation of MMP9, MMP2, and PCNA expression in VSMCs is indicative of increased cell proliferation and migration. We found that *γ*-GC inhibited the upregulation of these proteins in SHR-VSMCs but not in WKY-VSMCs. Similarly, *γ*-GC inhibited the mRNA levels of MMP9, MMP2, and PCNA in the aorta and mesenteric arteries from SHR, but not from WKY rats. Moreover, by down-regulating OPN and up-regulating *α*-SMA, *γ*-GC effectively prevented VSMC phenotypic switching and protected against vascular remodeling in SHR. Collectively, *γ*-GC selectively inhibited the pathological proliferation, migration, and phenotypic switching of SHR-VSMCs, highlighting its protective role in hypertensive vascular remodeling. While *γ*-GC significantly reduces MMP2 and MMP9 expression levels, further studies using gelatin zymography are warranted to confirm the corresponding changes in their functional proteolytic activity.

Oxidative stress, arising from an imbalance between ROS and the cellular antioxidant response, is a critical driver of hypertensive vascular remodeling [41]. While basal ROS serve physiological signaling functions, excessive ROS promote abnormal VSMC proliferation and migration [42]. As the major cellular antioxidant, GSH directly scavenges ROS or acts as a cofactor in the glutathione and thioredoxin systems [43]. Under oxidative stress conditions, GSH can also mediate protein S-glutathionylation, a reversible post-translational modification that conjugates GSH to cysteine residues, thereby modulating protein function and signaling pathways [44]. Indeed, RNA-Seq analysis revealed significant enrichment of oxidative stress-related pathways, and DCFH-DA and DHE fluorescence staining confirmed that *γ*-GC attenuated intracellular total ROS and superoxide anion levels in SHR-VSMCs, respectively. These findings indicate that *γ*-GC effectively alleviates oxidative stress in SHR-VSMCs.

Nrf2 protects cells against oxidative damage by upregulating antioxidant gene expression and maintaining intracellular redox homeostasis [11]. Our RNA-Seq results indicated that *γ*-GC significantly upregulated the expression of Nrf2 downstream antioxidant genes. Upon activation, Nrf2 translocates to the nucleus [45]. We observed that *γ*-GC increased nuclear Nrf2 protein levels and decreased cytosolic Nrf2 protein levels, indicating Nrf2 activation and enhanced antioxidant defense. To determine whether *γ*-GC inhibits vascular remodeling in hypertension through Nrf2 signaling, we employed both *in vitro* and *in vivo* rescue strategies. In SHR-VSMCs, the specific Nrf2 inhibitor ML385 remarkably reversed the *γ*-GC-induced inhibition of migration, proliferation, and phenotypic switching. Crucially, ML385 abolished the protective effects of *γ*-GC on blood pressure and vascular remodeling in SHR, confirming that the Nrf2 pathway is essential for the vascular benefits of *γ*-GC.

Keap1 acts as an inhibitor of Nrf2 by binding to it and preventing nuclear translocation [46]. Therefore, disrupting the Keap1 and Nrf2 interaction represents a novel strategy for activating Nrf2. Co-immunoprecipitation experiments revealed that *γ*-GC disrupts the Keap1-Nrf2 complex, facilitating Nrf2 nuclear accumulation. Initial molecular docking analyzes suggested a possible interaction between *γ*-GC and Keap1. However, thermodynamic considerations indicated that direct non-covalent binding was unlikely to competitively displace the high-affinity endogenous Nrf2-ETGE motif under physiological conditions. Given the role of *γ*-GC in elevating intracellular GSH levels, a more plausible mechanism involves Keap1 S-glutathionylation at reactive cysteine residues, which induces conformational changes that lead to Nrf2 release [35]. Non-reducing immunoblotting analysis confirmed increased Keap1 S-glutathionylation in *γ*-GC-treated SHR-VSMCs. Furthermore, pretreatment with the GSH synthesis inhibitor BSO abolished *γ*-GC-induced Nrf2 activation. Together, these findings demonstrate that *γ*-GC inhibits SHR-VSMC migration, proliferation, and phenotypic switching by promoting Keap1 S-glutathionylation and the consequent activation of the Nrf2 signaling pathway. Importantly, this highlights a novel pharmacological role for *γ*-GC. Beyond acting as a passive precursor for direct ROS scavenging, *γ*-GC functions as an active signaling molecule that couples the cellular redox state to Nrf2-driven vascular protection via specific covalent protein modification.

Notably, the systemic antihypertensive effects of *γ*-GC involve integrated antioxidant actions across multiple physiological systems. By efficiently elevating intracellular GSH levels, *γ*-GC has been demonstrated to restore systemic redox homeostasis and suppress inflammatory responses [20], which are crucial prerequisites for maintaining endothelial function and vascular tone. Although the current study primarily focused on the arterial media and did not directly assess the specific oxidative status of extra-vascular organs, the overall reduction in blood pressure suggests a broader systemic benefit. Furthermore, considering that oxidative stress in the kidneys and central nervous system is a primary driver of hypertension [47], *γ*-GC may also exert systemic effects by alleviating extra-vascular oxidative damage or modulating sympathetic outflow. These multi-organ antioxidant actions likely complement the media-specific Keap1/Nrf2 signaling, together contributing to the overall therapeutic potential of *γ*-GC.

Taken together, our study demonstrates that *γ*-GC is a potent and selective modulator of vascular remodeling. However, two methodological limitations should be noted: continuous blood pressure monitoring via wireless telemetry was not utilized, and acute vascular functional data (e.g. relaxation assays) were not included, as our focus remained on structural remodeling. Both represent important avenues for future investigations. Finally, as a dipeptide, the oral bioavailability of *γ*-GC may be restricted by gastrointestinal degradation. Nevertheless, its excellent *in vivo* safety profile and potent target-organ protection strongly support its long-term translational potential. Future development of advanced oral delivery systems will be key to overcoming this barrier and paving the way for its clinical application in chronic hypertension.

## Conclusion

In summary, the present study demonstrates a potent protective role of *γ*-GC in hypertension-associated vascular remodeling. We found that *γ*-GC selectively reduces blood pressure and attenuates vascular remodeling in SHR, without affecting normotensive WKY rats. Mechanistically, *γ*-GC suppresses VSMC migration, proliferation, and phenotypic switching through the activation of the Keap1/Nrf2 signaling pathway, which is uniquely mediated by the promotion of Keap1 S-glutathionylation. Overall, our data position *γ*-GC as a promising and safe therapeutic candidate for the management of hypertension and its associated vascular complications.

## Supplementary Material

supplementary.doc

Original western blots.pdf Original western blots.pdf

ARRIVE guidelines Author Checklist.pdf ARRIVE guidelines Author Checklist.pdf

## Data Availability

The data supporting the findings of this study are available from the corresponding author on reasonable request. RNA-sequence data can be accessed freely on Figshare repository (https://doi.org/10.6084/m9.figshare.30909203).
