# Supplementary material for: γ-Glutamylcysteine alleviates hypertension and vascular remodeling in SHR by suppressing oxidative stress through Keap1 S-glutathionylation
Source: Redox Rep. 2026 Sep 22;31(1):2734676. doi: 10.1080/13510002.2026.2734676 (PMC13600300; doi:10.1080/13510002.2026.2734676)

original western blots

Figure 2E

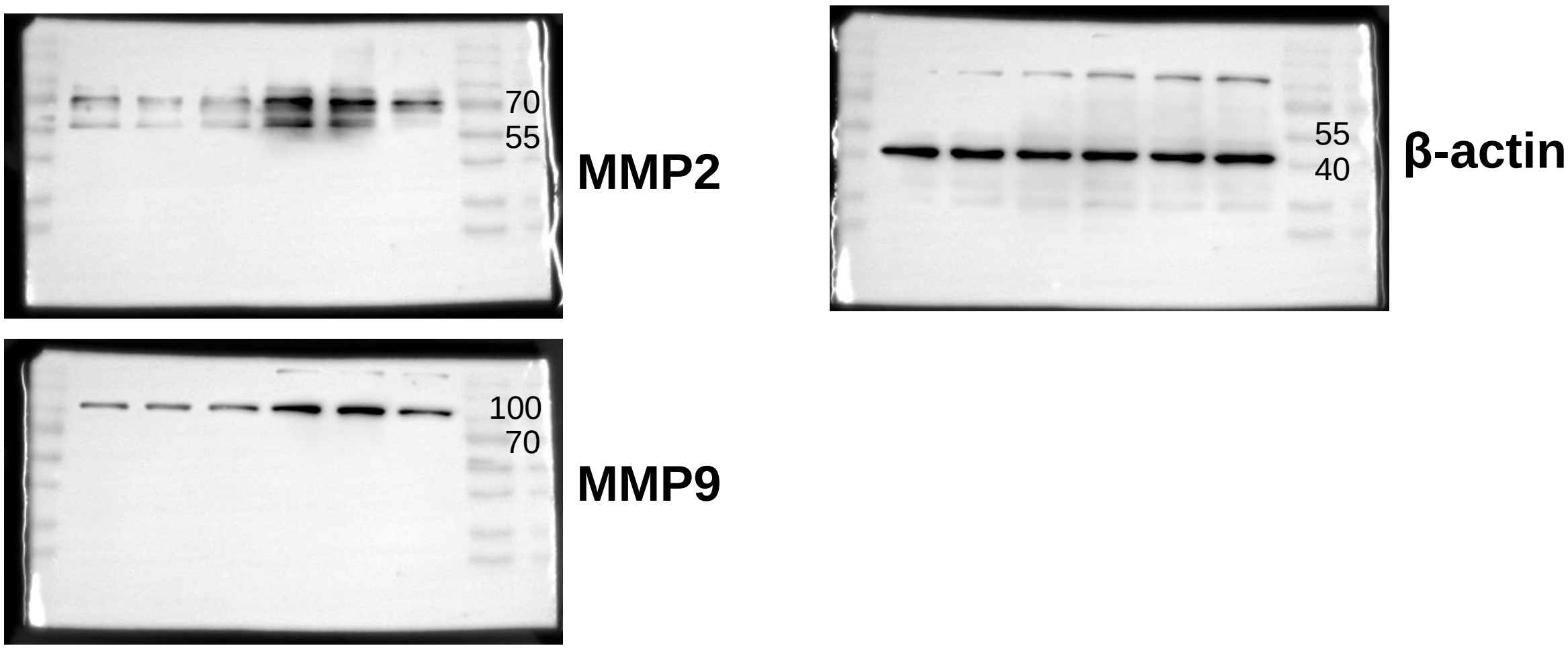

Figure 3A

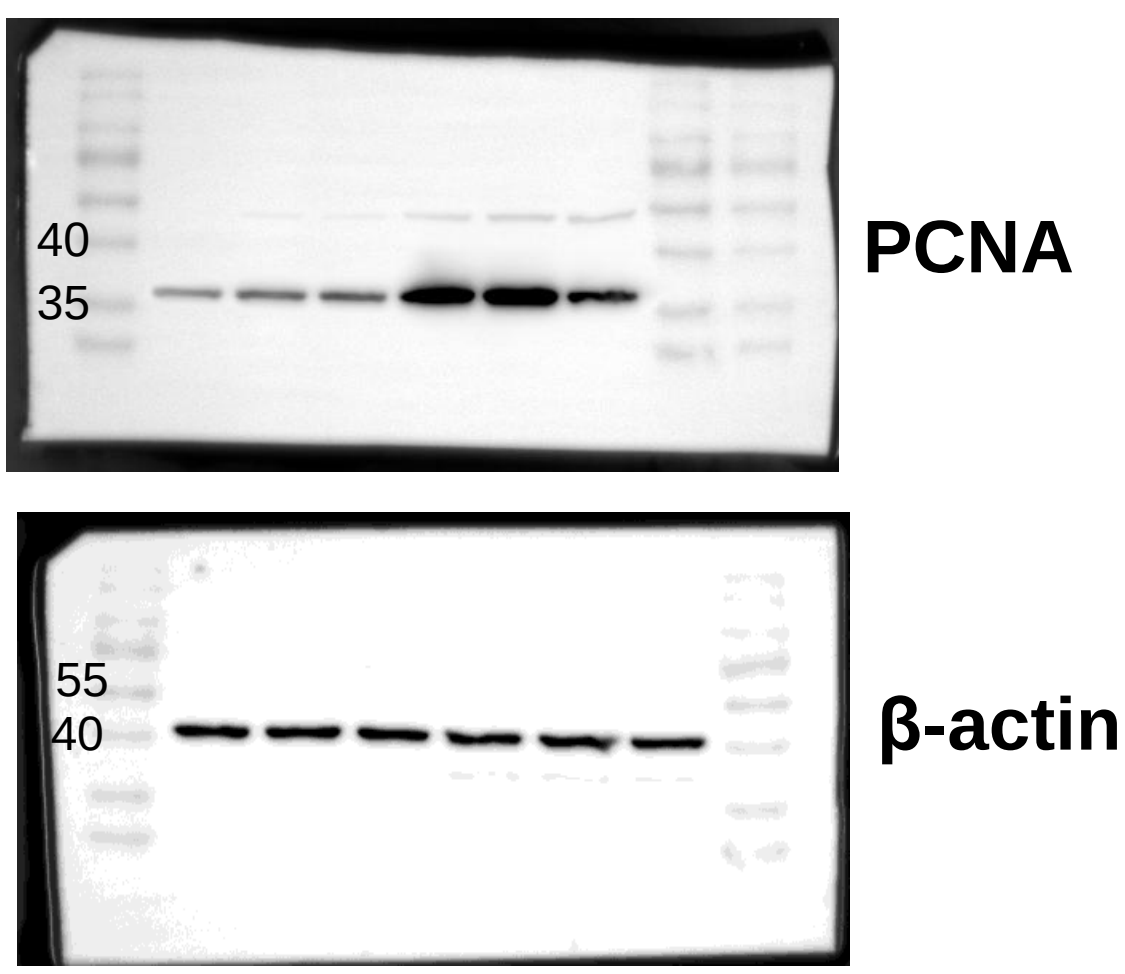

Figure 3E

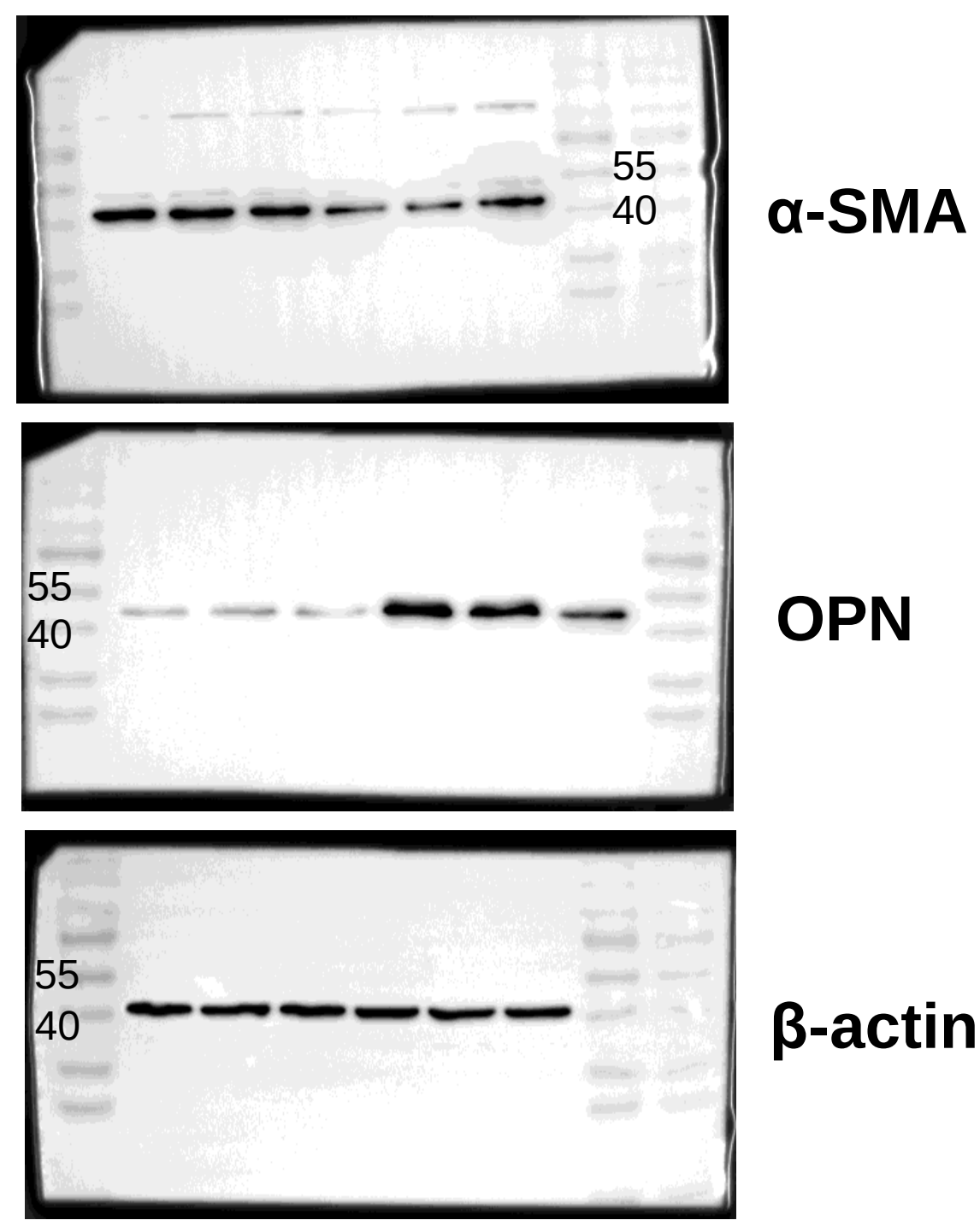

Figure 5C

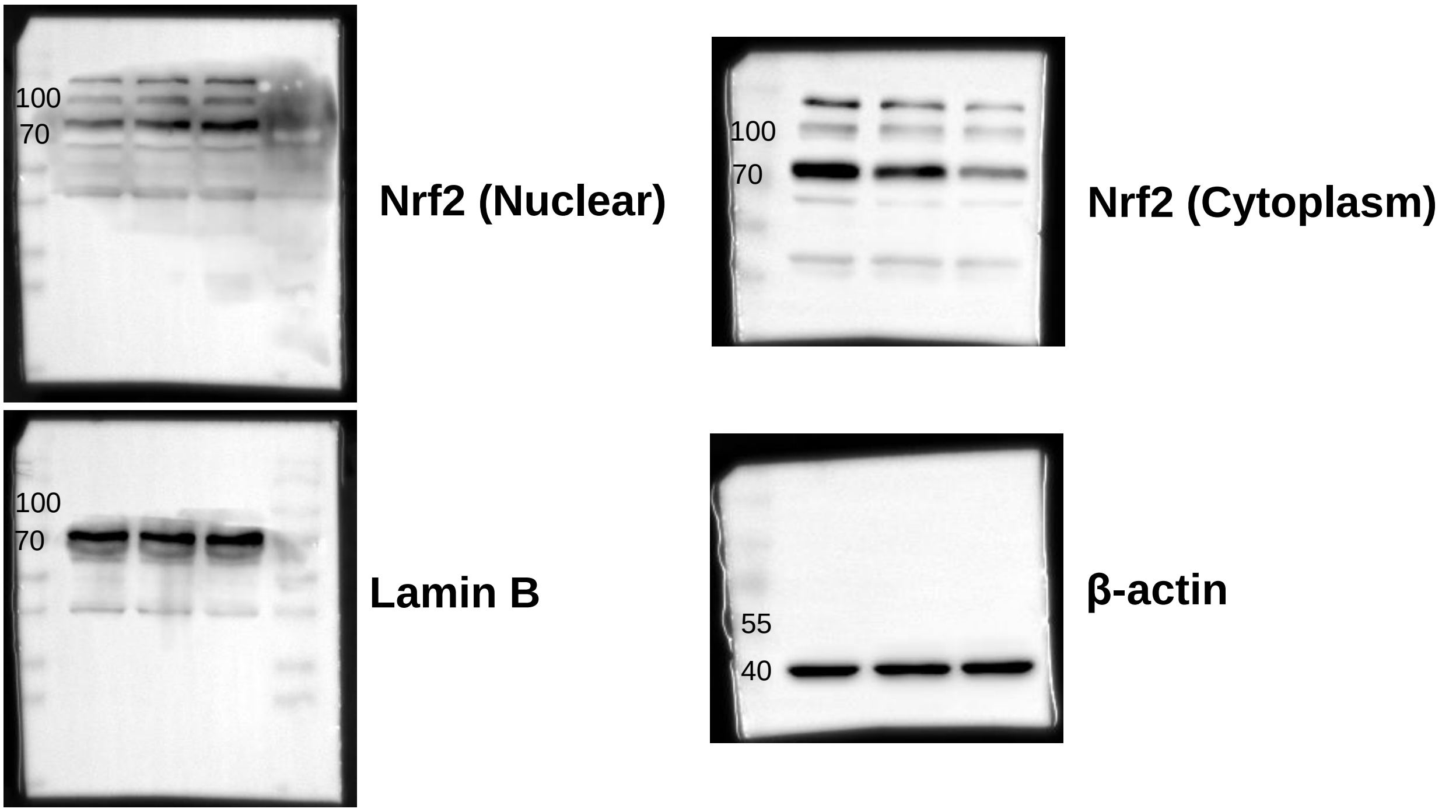

Figure 6E

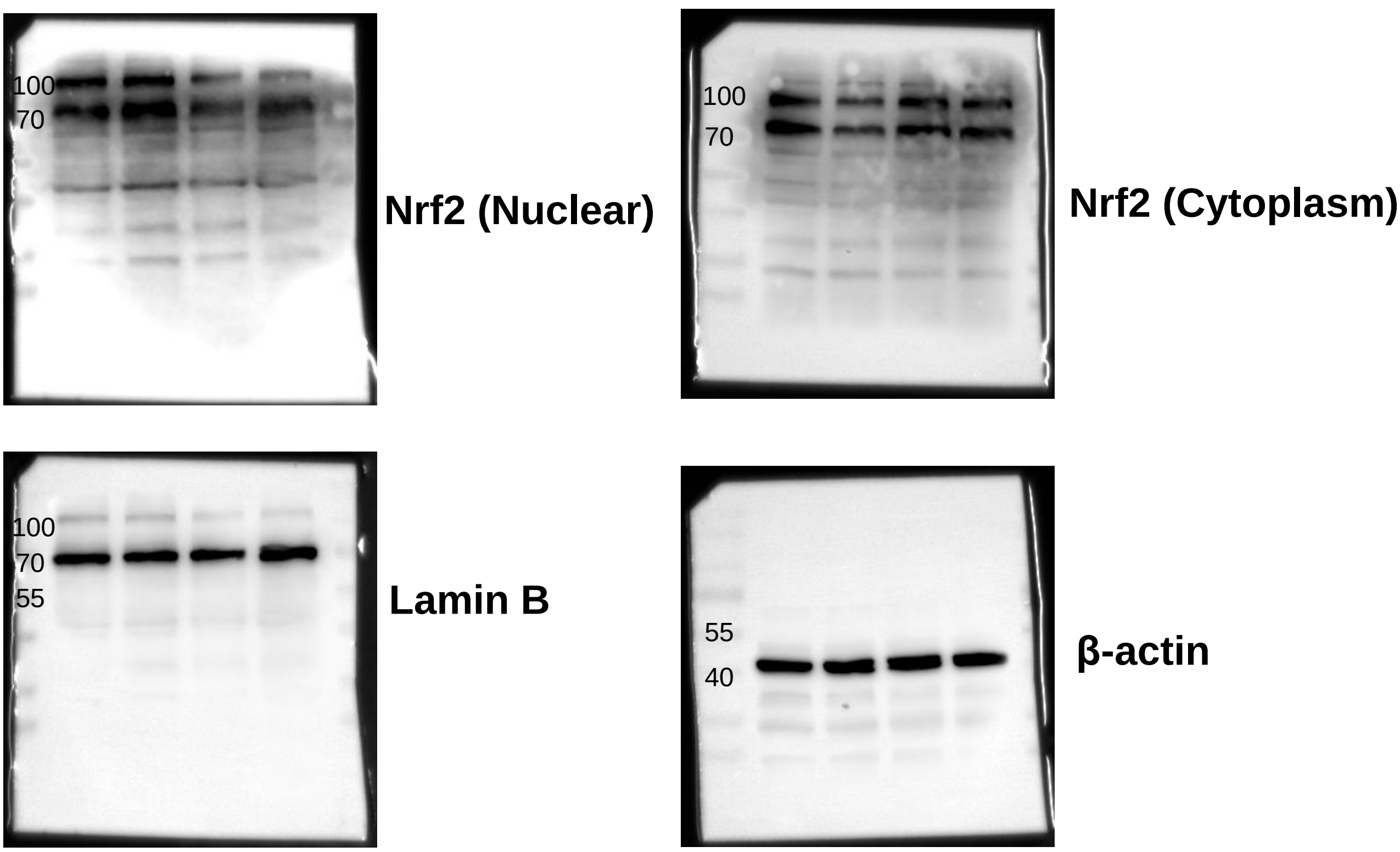

Figure 7C

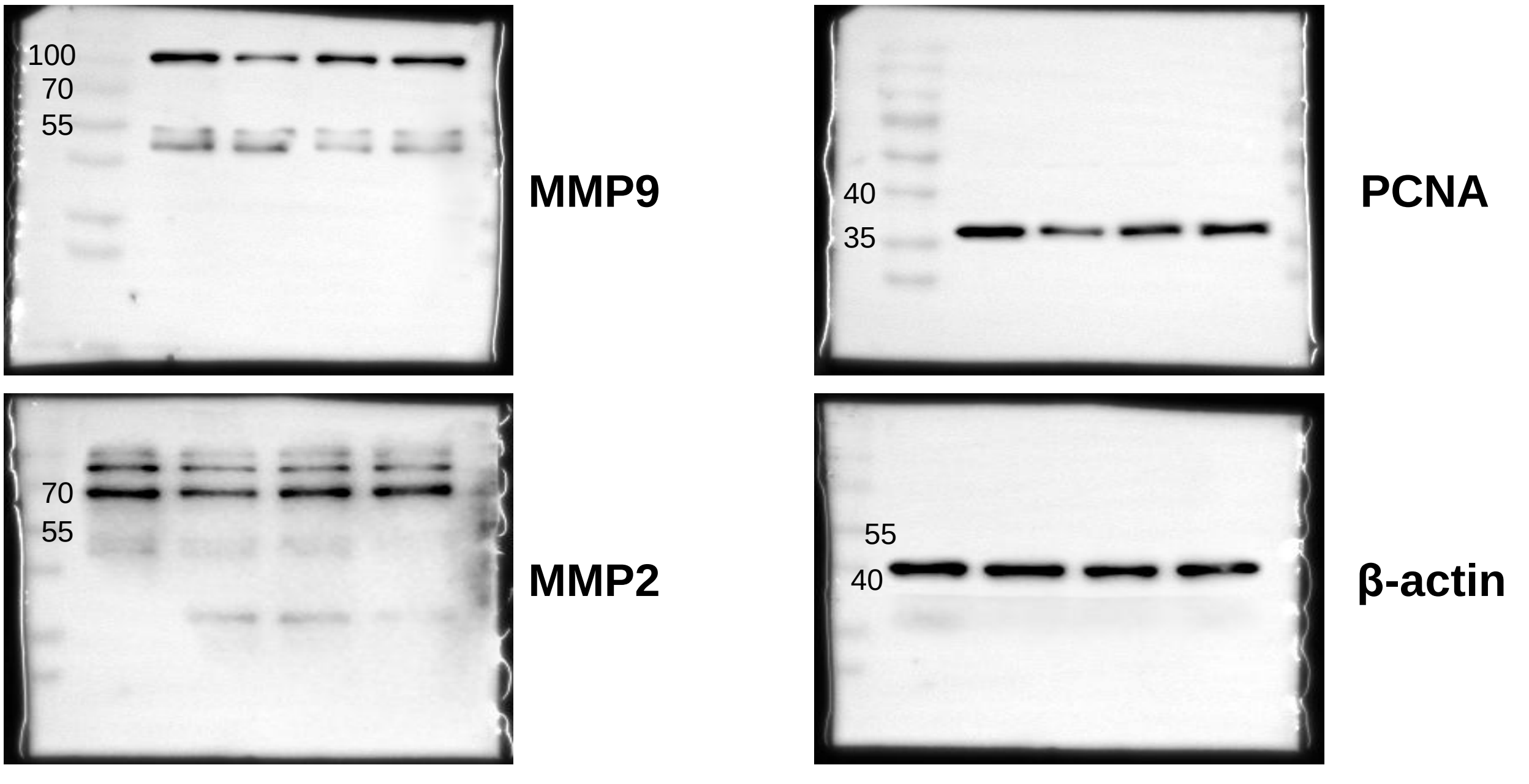

Figure 8A

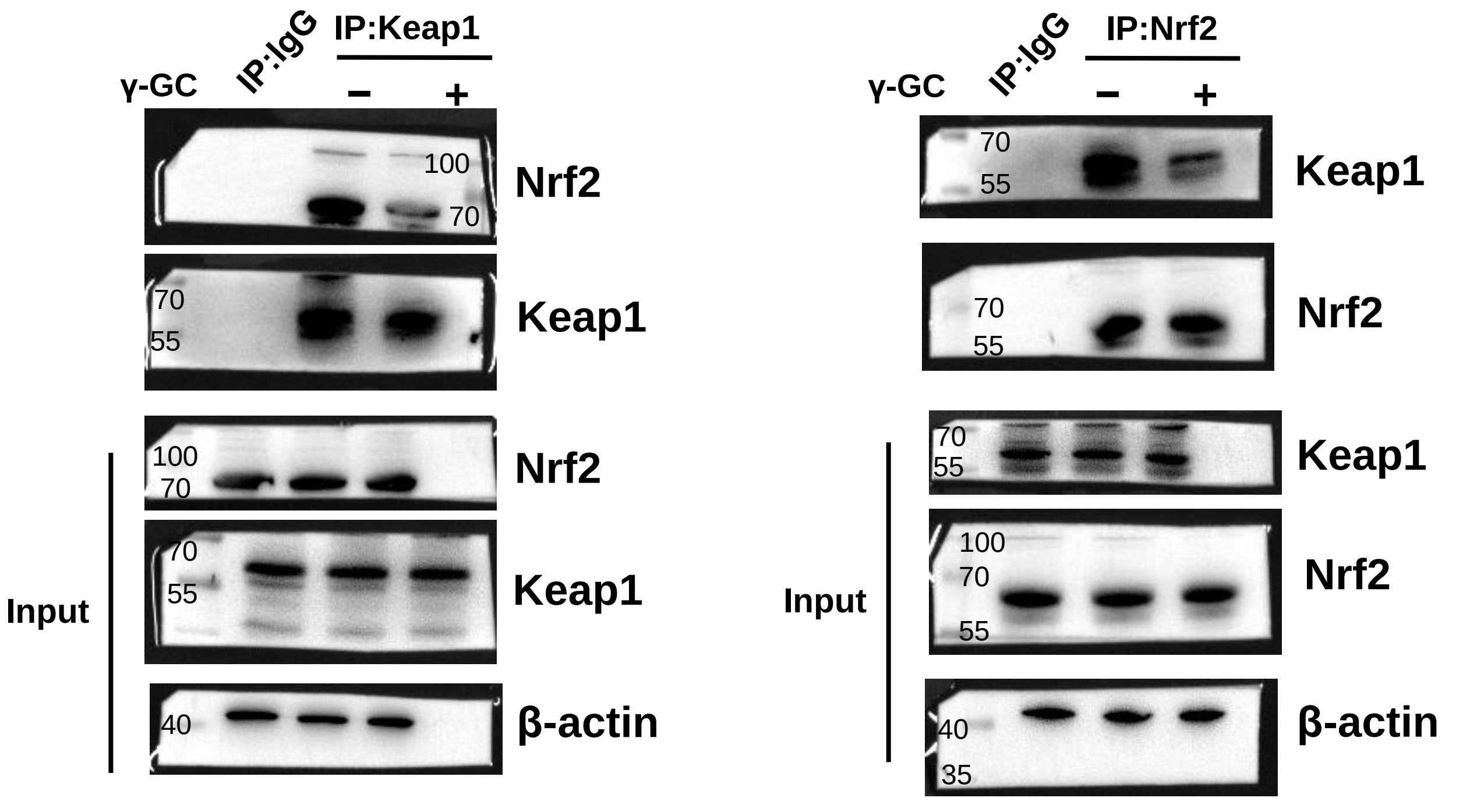

Figure 8B

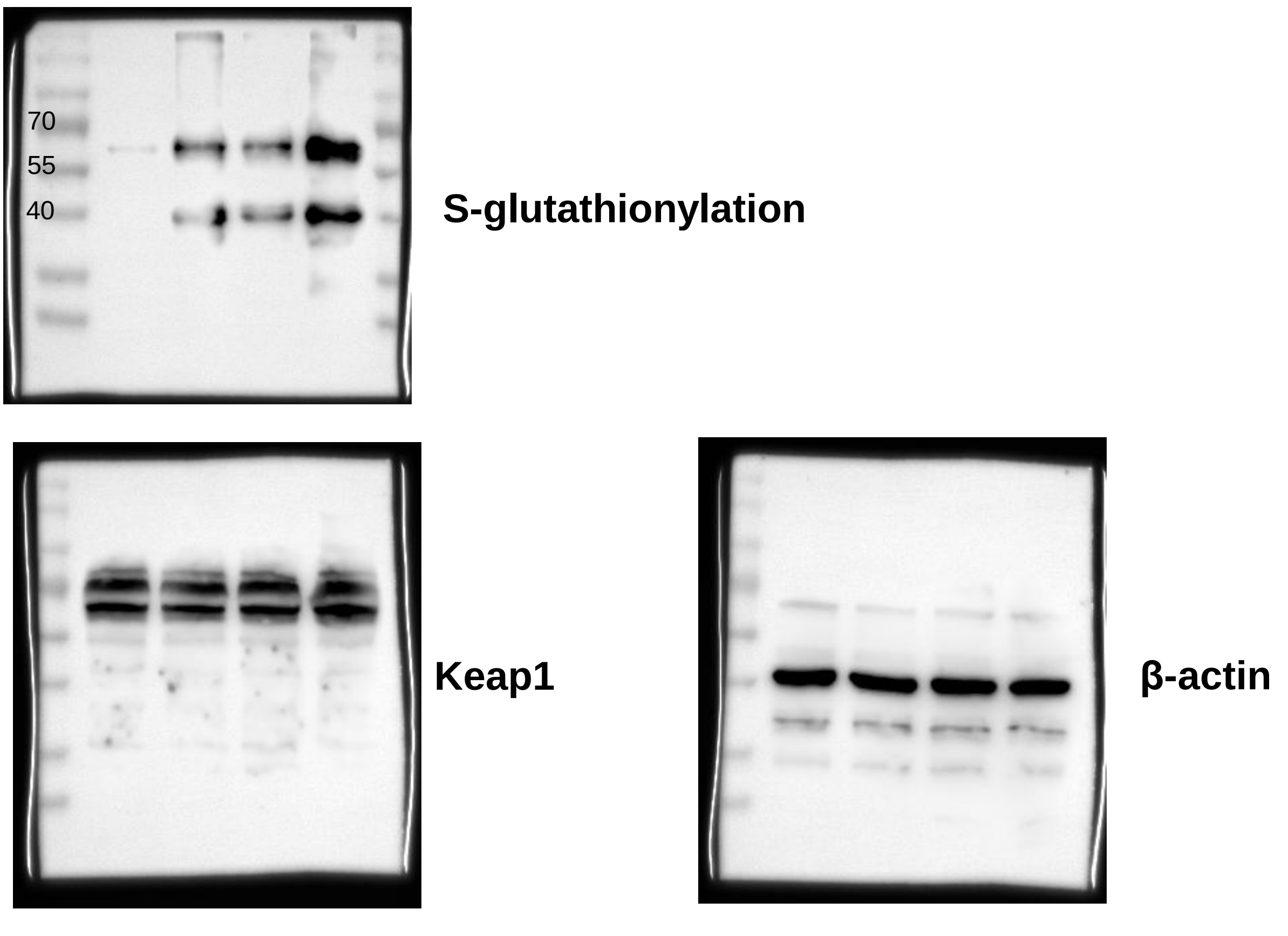

Figure 8D

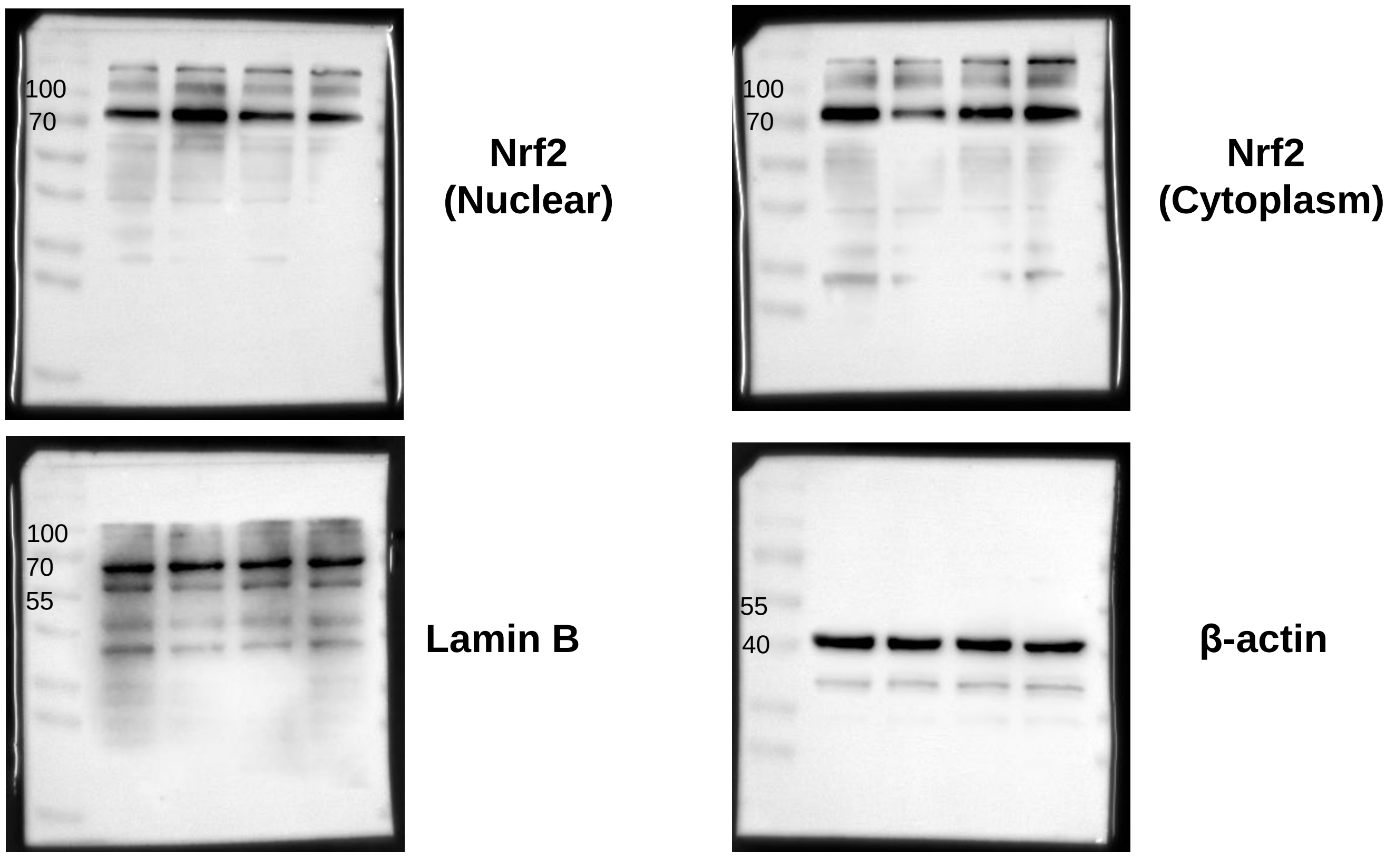

Figure S7A

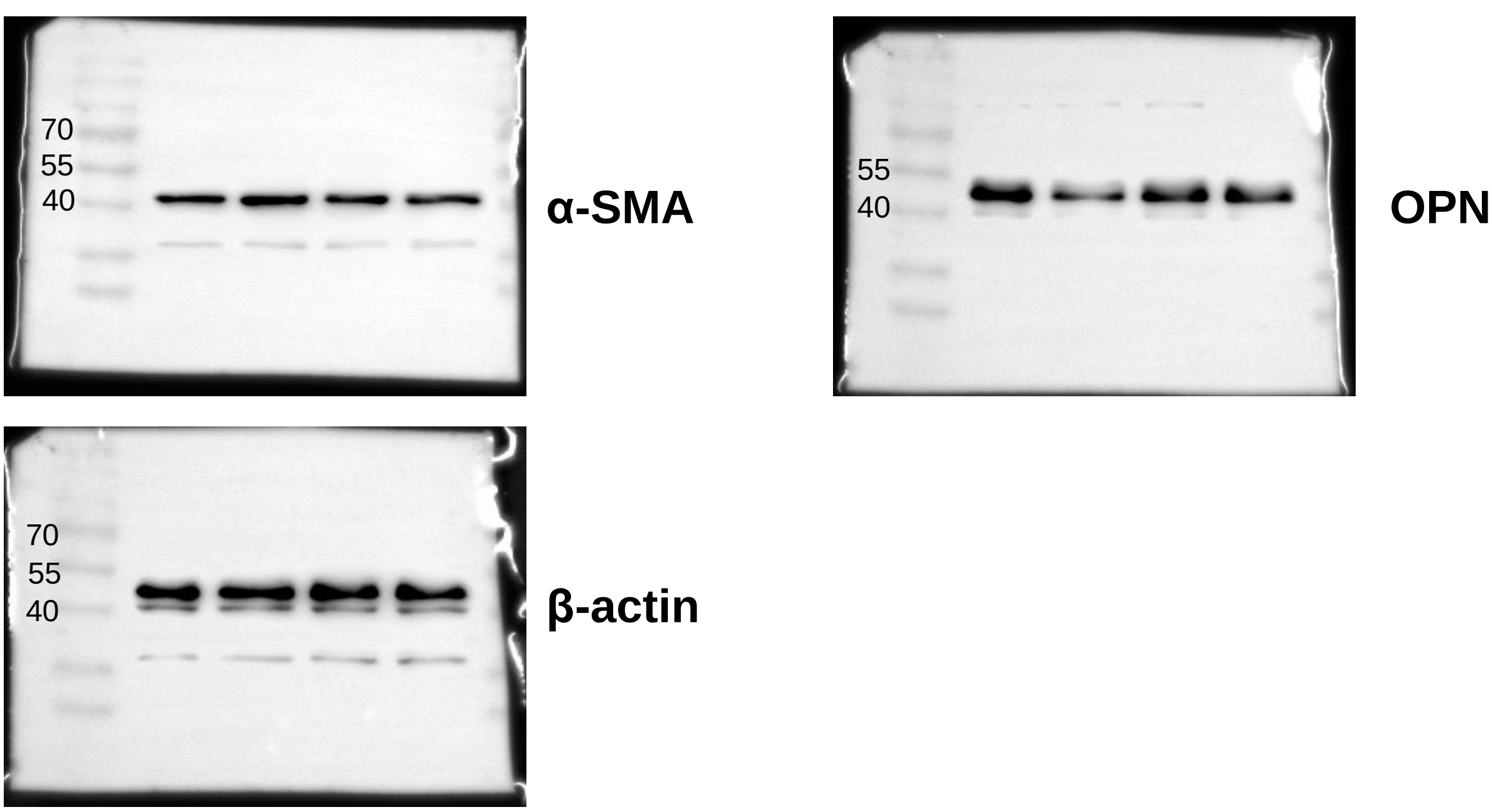

Supplement: Supplementary file 1 [file YRER_A_2734676_SM2688.pdf]
