# Supplementary material for: γ-Glutamylcysteine alleviates hypertension and vascular remodeling in SHR by suppressing oxidative stress through Keap1 S-glutathionylation
Source: Redox Rep. 2026 Sep 22;31(1):2734676. doi: 10.1080/13510002.2026.2734676 (PMC13600300; doi:10.1080/13510002.2026.2734676)
Supplement: Supplementary file 3 [file YRER_A_2734676_SM2690.doc]

**Supplementary Information**

**Supplementary Figures**

**Figure S1. γ-GC does not affect the viability of VSMCs from SHR and WKY rats**

Cell viability was determined using the CCK-8 Assay Kit. Primary aortic VSMCs from WKY incubated with different doses (0-160 μM) of γ-GC for 24 h (**A**) or 48 h (**B**). Primary aortic VSMCs from SHR incubated with different doses (0-160 μM) of γ-GC for 24 h (**C**) or 48 h (**D**). Data are mean ± SD. N = 6 per group. **P*<0.05, ***P*<0.01 vs indicated control. **A**, **C** and **D** were analyzed by one-way ANOVA followed by Tukey's post hoc test. **B** were analyzed by the Kruskal-Wallis test followed by Dunn's multiple comparisons test.

**Figure S2. γ-GC reduces arterial blood pressure in SHR but not in WKY rats**

(**A**) Schematic diagram showing the protocol of γ-GC intraperitoneal injection (100 mg/kg, every 2 days for 4 weeks) and blood pressure measurements in SHR and WKY rats. (**B**) SHR and WKY rats mean arterial pressure (MAP) and heart rate (HR) were measured with the carotid catheter method. Data are mean ± SD. N = 6 per group. **P*<0.05 vs indicated control. **B** was analyzed by one-way ANOVA followed by Tukey's post hoc test.

**Figure S3. γ-GC attenuates vascular remodeling in SHR but not in WKY rats**

(**A-B**) Bar graphs showing the Masson’s staining analysis for lumen diameter in the aorta and MA from SHR and WKY rats. (**C-D**) The relative expression levels of *PCNA* mRNA in SHR and WKY rat aortas and MAs were measured by qPCR. Data are mean ± SD. N = 6 per group. ***P*<0.01 vs indicated control. **A**-**D** were analyzed by one-way ANOVA followed by Tukey's post hoc test.

**Figure S4.** **Safety evaluation of γ-GC in WKY and SHR rats**

SHR and WKY rats were treated with γ-GC (100 mg/kg) or saline via intraperitoneal injection every 2 days for 4 weeks. (**A**) Changes in body weight of rats in different groups. (**B-E**) Serum levels of ALT, AST, BUN, and SCr. (**F**) Representative H&E staining images of liver and kidney sections are shown. Data are mean ± SD. N = 6 per group in **A**-**E**. N = 3 per group in **F**. ***P*<0.01 vs indicated control. **A**-**E** were analyzed by one-way ANOVA followed by Tukey's post hoc test.

**Figure S5. γ-GC does not influence ROS levels in WKY-VSMCs**

WKY-VSMCs were treated with PBS or different concentrations of γ-GC (40 or 80 μM). (**A**) The levels of superoxide anion were detected by DHE fluorescence staining. (**B**) The levels of reactive oxygen species (ROS) analyzed using the DCFH-DA probe. Data are mean ± SD. N = 6 per group. **A**-**B** were analyzed by one-way ANOVA followed by Tukey's post hoc test.

**Figure S6.** **ML385 inhibits Nrf2 downstream targets and reverses the effect of γ-GC on vascular remodeling in SHR.**

(**A-B**) The mRNA levels of *Hmox1* and *Nqo1* in the aortas were measured at the 4-week endpoint to confirm the sustained *in vivo* efficacy of ML385. Bar graphs showing the Masson’s staining analysis for lumen diameter (**C-D**) and the ratio of medial thickness to lumen diameter (**E-F**) in the aorta and MA from SHR. Data are mean ± SD. N = 6 per group. ***P*<0.01 vs indicated control. **A** and **C**-**F** were analyzed by one-way ANOVA followed by Tukey's post hoc test. **B** were analyzed by the Brown-Forsythe ANOVA followed by Dunnett’s post hoc test.

**Figure S7 ML385 reverses the effect of γ-GC on VSMC** **phenotypic switching**

SHR-VSMCs were pre-treated with ML385 (2 μM) and then were treat with PBS or γ-GC (80 μM). (**A**) The expression levels of VSMC contractile phenotype protein α-SMA and VSMC synthetic phenotype protein Osteopontin (OPN) were examined by immunoblotting. (**B**) The relative expression levels of *α-SMA* and *OPN* mRNA were measured by qPCR. Data are mean ± SD. N = 4 per group in **A**. N = 6 per group in **B**. ***P*<0.01 vs indicated control. **A**-**B** were analyzed by one-way ANOVA followed by Tukey's post hoc test.

**Figure S8 Molecular docking of γ-GC with Nrf2 and Keap1.**

(**A**) 3D structures showing the Molecular docking of γ-GC (yellow) and Nrf2 (orange) and Keap1 (green). (**B**) Identification of non-covalent interactions between γ-GC and Keap1.

**Figure S9 BSO reverses the γ-GC-induced increase in the GSH/GSSG ratio**

SHR-VSMCs were pre-treated with BSO (1 mM) and then treated with PBS or γ-GC (80 μM). Vehicle (Veh) served as a control. The GSH/GSSG ratio was measured using a commercial assay kit according to the manufacturer’s instructions. Data are mean ± SD. N = 6 per group. ***P*<0.01 vs indicated control. Data were analyzed by one-way ANOVA followed by Tukey's post hoc test.

**Supplementary Tables**

**Table S1.** Antibodies

| Target antigen | Vendor or Source | Catalog # | Working concentration | Lot # (preferred but not required) |
| --- | --- | --- | --- | --- |
| β-actin Polyclonal Antibody | Proteintech | 20536-1-AP | 0.2 μg/mL | 2022033148 |
| β-actin Monoclonal Antibody | Abways | AB0035 | 1 μg/mL | F210085 |
| PCNA Polyclonal Antibody | Bioworld | BS1289 | WB: 1 μg/mL  IHC: 10 μg/mL | AA33161 |
| MMP9 (N-terminal) Polyclonal Antibody | Proteintech | 10375-2-AP | 0.4 μg/mL | 00023850 |
| MMP2 Polyclonal Antibody | Proteintech | 10373-2-AP | 0.65 μg/mL | 00026123 |
| Osteopontin Polyclonal Antibody | Proteintech | 25715-1-AP | 0.4 μg/mL | 00024375 |
| Smooth Muscle Actin Polyclonal Antibody | Proteintech | 14395-1-AP | 0.5 μg/mL | 00044853 |
| Nrf2 Polyclonal Antibody | Proteintech | 16396-1-AP | WB: 0.8 μg/mL  IF: 8 μg/mL | 00183744 |
| Keap1 Polyclonal Antibody | Proteintech | 10503-2-AP | WB: 0.6 μg/mL  IP: 6 μg/mL | 00172150 |
| Lamin B1 Monoclonal Antibody | Abways | AB0054 | 1 μg/mL | F154405 |
| Glutathione Monoclonal Antibody | Virogen | #101-A-100 | 1 μg/mL | GS-D8/G2-84 |

**Table S2. Primer sequences for PCR**

| Target genes | Oligonucleotide primers (5’-3’) | Product length (bp) | NCBI Accession Number |
| --- | --- | --- | --- |
| Rat *Gstp1* | FP:TGGAGGACCTTCGATGCAAA  RP:AGCAGGTCCAGCAAGTTGTA | 190 | NM_012577.2 |
| Rat *α-SMA* | FP:ACTGGGACGACATGGAAAAG  RP:CATCTCCAGAGTCCAGCACA | 240 | NM_031004.2 |
| Rat *OPN* | FP:TGAGTTTGGCAGCTCAGAGG  RP:TCGTCGTCGTCATCATCGTC | 210 | NM_012881.2 |
| Rat *MMP2* | FP:GTCCTGACCAAGGATATAGCC  RP:AGACCCAGTACTCATTCCCTG | 465 | NM_031054.2 |
| Rat *MMP9* | FP:GATCCCCAGAGCGTTACTCG  RP:GTTGTGGAAACTCACACGCC | 132 | NM_031055.2 |
| Rat *PCNA* | FP:TTTGAGGCACGCCTGATCC  RP:GGAGACGTGAGACGAGTCCAT | 135 | NM_022381.3 |
| Rat *β-actin* | FP:CTGAGAGGGAAATCGTGCGT  RP:CTTCTCCAGGGAGGAAGAGGA | 104 | NM_031144.3 |
| Rat *Sqstm1* | FP:ACGACTGGACGCATTTGTCT  RP:TGGTGGGAGATGTGGGTACA | 158 | NM_175843.5 |
| Rat *Nqo1* | FP:GATTGTATTGGCCCACGCAG  RP:GATTCGACCACCTCCCATCC | 99 | NM_017000.3 |
| Rat *Txn1* | FP:CAAATGCATGCCGACCTTCC  RP:GTGGCTTCGAGCTTTTCCTTG | 87 | NM_053800.3 |
| Rat *Hmox1* | FP:TTTTCACCTTCCCGAGCAT  RP:GCCTCTTCTGTCACCCTGT | 112 | NM_012580.2 |

**Table S3. Baseline characteristics of rat models**

| Parameters | Age (wk) | HW (g) | BW (g) | HW/BW (mg/g) | SBP (mmHg) | n |
| --- | --- | --- | --- | --- | --- | --- |
| WKY | 12 | 1.06±0.03 | 306.9±3.9 | 3.47±0.12 | 126.6±2.4 | 6 |
| SHR | 12 | 1.14±0.02 | 302.3±3.7 | 3.79±0.08 | 175.4±3.7** | 6 |

HW: heart weight; BW: body weight; SBP: systolic blood pressure. ***p*<0.01 vs WKY, unpaired Student's *t* test.
